# ELK3 destabilization by speckle-type POZ protein suppresses prostate cancer progression and docetaxel resistance

**DOI:** 10.1038/s41419-024-06647-0

**Published:** 2024-04-17

**Authors:** Cheol-Jung Lee, Heejung Lee, Seo Ree Kim, Soo-Bin Nam, Ga-Eun Lee, Kyeong Eun Yang, Guk Jin Lee, Sang Hoon Chun, Han Chang Kang, Joo Young Lee, Hye Suk Lee, Sung-Jun Cho, Yong-Yeon Cho

**Affiliations:** 1 BK21-4th Team, College of Pharmacy, The Catholic University of Korea, 43, Jibong-ro, Wonmi-gu, Bucheon-si, Gyeonggi-do 14662 Korea; 2 Biopharmaceutical research center, Ochang Institute of Biological and Environmental Science, Korea Basic Science Institute (KBSI), 162, Cheongju, 28119 Korea; 3 Department of Hospital Pathology, Bucheon St. Mary’s Hospital, College of Medicine, The Catholic University of Korea, Seoul, Korea; 4 Division of Medical Oncology, Department of Internal Medicine, Bucheon St. Mary’s Hospital, College of Medicine, The Catholic University of Korea, Seoul, Korea; 5 RCD Control·Material Research Institute, The Catholic University of Korea, 43, Jibong-ro, Wonmi-gu, Bucheon-si, Gyeonggi-do 14662 Republic of Korea; 6 University of Minnesota Department of Medicine, 420, Delaware St., SE, Minneapolis, MN MN55455 USA

**Keywords:** Ubiquitylation, Oncogenesis

## Abstract

Accumulating evidence demonstrates that the activity regulation of ELK3, a member of the E26 transformation-specific oncogene family, is critical to regulating cell proliferation, migration, and survival in human cancers. However, the molecular mechanisms of how ELK3 induces chemoresistance in prostate cancer (PCa) have not been elucidated. In this study, we found that SPOP and ELK3 are an interacting partner. The interaction between SPOP and ELK3 resulted in increased ELK3 ubiquitination and destruction, assisted by checkpoint kinase-mediated ELK3 phosphorylation. Notably, the modulation of SPOP-mediated ELK3 protein stability affected the c-Fos-induced cell proliferation and invasion of PCa cells. The clinical involvement of the SPOP-ELK3 axis in PCa development was confirmed by an immunohistochemical assay on 123 PCa tissues, with an inverse correlation between increased ELK3 and decreased SPOP being present in ~80% of the specimens. This observation was supported by immunohistochemistry analysis using a SPOP-mutant PCa specimen. Finally, docetaxel treatment induced cell death by activating checkpoint kinase- and SPOP-mediated ELK3 degradation, while SPOP-depleted or SPOP-mutated PCa cells showed cell death resistance. Notably, this observation was correlated with the protein levels of ELK3. Taken together, our study reveals the precise mechanism of SPOP-mediated degradation of ELK3 and provides evidence that SPOP mutations contribute to docetaxel resistance in PCa.

## Introduction

Prostate cancer (PCa) is the most commonly diagnosed cancer in Western males and the second leading cause of death among men with cancers [1]. Androgen deprivation therapy is an effective therapeutic approach for PCa [2], but many patients show significant resistance to treatment and progress to metastatic castration-resistant PCa [3]. Docetaxel is an anticancer drug that demonstrated improvements in the overall survival of these patients in a large-scale randomized clinical study (TAX 32713) in 2004; thus, this drug is the current standard treatment for metastatic castration-resistant PCa [4]. Mechanistically, docetaxel interrupts the depolymerization of microtubules by binding to the β-tubulin subunit and inhibiting the gene expression of antiapoptotic genes, such as *bcl-2* and *bcl-xL*, resulting in cell cycle arrest and apoptosis [5, 6]. In addition, several clinical studies (CHAARTED, GETUG-AFU 15, and STAMPEDE) conducted in 2005 showed that combination treatment with androgen deprivation therapy and docetaxel significantly improved the overall survival of metastatic hormone-sensitive PCa patients [7]. Despite the survival benefit of docetaxel, almost all patients treated with docetaxel encounter drug resistance [8]. Therefore, it is essential to elucidate the molecular mechanism that induces docetaxel resistance in PCa.

ELK3 (known as Net, SAP-2, or Erp) is a family member of the E26 transformation-specific transcription factors and forms a ternary complex with a serum response factor to control the expression of target genes [9], such as *c-fos* [9]. Emerging evidence has demonstrated that ELK3 participates and acts as a critical oncoprotein in human cancers, including glioma, melanoma, gastric cancer, breast cancer, and PCa. For example, ELK3 increases cell migration and the invasion of gastric cancer through the regulation of extracellular matrix remodeling-associated genes [10]. Moreover, the upregulation of ELK3 expression by LINC00662, a noncoding RNA, promotes melanoma cell proliferation, migration, and invasion [11]. In PCa, ELK3 knockdown is involved in the arrest of the S and M cell cycle phases and leads to the inhibition of cell growth and migration [12]. Our research group also reported that ELK3 activation mediated by RSK2 induces the transformation of JB6 Cl41 cells and the proliferation of breast cancer cells via regulation of *c-fos* gene expression [13]. However, although the important role of ELK3 in tumor progression is well-known, the molecular mechanisms involved in the regulation of ELK3 protein degradation through ubiquitination remain largely unknown.

Speckle-type POZ protein (SPOP) is an adaptor protein of Cullin3 (CUL3)-based ubiquitin ligase complexes. SPOP possesses substrate selectivity through the N-terminal Merprin and TRAF homology (MATH) domain and interacts with CUL3 via the C-terminal Broad-complex, Tramtrack, and Bric-a-brac (BTB) domain [14]. SPOP has been identified as a substrate-recruiting component for various proteins, including androgen receptor (AR) [15], double-stranded DNA-binding Ets-like Kruppel-associated box protein [16], steroid receptor coactivator 3 [17], bromodomain-containing protein 4 (BRD4) [18], 3-phosphoinositide-dependent protein kinase-1 [19], and caprin 1 [20]. The identification of various substrates targeted by SPOP suggests that this protein may play a critical role in nuclear chromatin remodeling-mediated gene expression. These substrates are involved in diverse cellular processes, including gene regulation, protein stability, and cell signaling. Notably, many of these substrates have been linked to oncogenic processes, implying that SPOP might function as a tumor suppressor. Moreover, recurrent somatic mutations of the *SPOP* gene are identified in up to 15% of PCa cases [21, 22], and the mutations are clustered in the MATH domain [23], which leads to accumulating oncogenic substrates and accelerated PCa progression via increased cell proliferation, migration, and invasion [24, 25]. However, none of the biological roles or molecular mechanisms of the SPOP-ELK3 stability regulation pathway have been elucidated.

In this study, we demonstrated that ELK3 plays a crucial role in PCa progression. We confirm that the ELK3 protein level is elevated in PCa tissue compared to normal tissue. Importantly, we reveal precise mechanisms of ELK3 protein stability via the ubiquitin-proteasome system. SPOP promotes ELK3 ubiquitination and destruction, triggered by checkpoint kinase-1/2 (CHK1/2)-mediated ELK3 phosphorylation. Notably, docetaxel induces CHK1/2 activity, resulting in ELK3 degradation. Furthermore, PCa-derived mutations in *SPOP* fail to induce ELK3 degradation and docetaxel-induced cell death. Thus, our results provide a potential new therapeutic insight for docetaxel-resistant PCa.

## Materials and methods

General methodologies, including cell proliferation and viability assays, immunoblotting and immunoprecipitation, luciferase reporter assay, and migration assay, employed in this study are described in detail in Supplemental materials. Material sharing is available by contacting with corresponding author (YY Cho, yongyeon@catholic.ac.kr).

### Antibodies

In general, total antibodies were used at a 1:2000 dilution in 5% skim milk in 1× phosphate-buffered saline (PBS), and phosphoantibodies were used at a 1:1000 dilution in 3% bovine serum albumin (BSA) in 1× PBS for western blotting (WB). Anti-ELK3 (cat. no.: NBP2-01264) antibody was purchased from Novus (St. Louis, MO, USA). The anti-SPOP antibody was purchased from Proteintech (cat. no.: 16750-1-AP, Rosemont, IL, USA). Anti-HA agarose beads were purchased from Sigma-Aldrich (cat. no.: A2095, St Louis, MO, USA). Anti-Xpress (cat. no.: R910-25) antibody was purchased from Thermo Fisher Scientific (Waltham, MA, USA). Anti-Flag (cat. no.: M185-3), anti-Flag-HRP (cat. no.: M185-7), and anti-HA-HRP (cat. no.: M180-7) antibodies were purchased from MBL. Anti-c-Myc (cat. no.: SC-40) and β-actin (cat. no.: SC-47778) antibodies were purchased from Santa Cruz Biotechnology (Dallas, TX, USA). Anti-Cul3 (cat. no.: 2759), anti-pT68-CHK2 (cat. no.: 2197), anti-CHK2 (cat. no.: 6334), anti-pS345-CHK1 (cat. no.: 2348), anti-CHK1 (cat. no.: 2360), Cyclin E (cat. no.: 4129), anti-phospho-Akt substrate (cat. no.: 10001), anti-c-Fos (cat. no.: 2250) antibodies were purchased from Cell Signaling Technology (Danvers, MA, USA).

### Cell culture and treatment

All cells purchased from ATCC were maintained in the recommended cell culture media at 37 °C in a 5% CO_2_ humidified atmosphere. Specifically, HEK293T, HeLa, DU145, and 22Rv1 cells were cultured in Dulbecco’s modified Eagle’s medium (Thermo Fisher Scientific), while PC-3 cells were cultured in RPMI-1640 (Thermo Fisher Scientific) supplemented with L-glutamine. All media were supplemented with 10% fetal bovine serum (FBS) and 1% penicillin–streptomycin. Cells were treated with various compounds, including 10 μM MG132 (cat. no.: C2211, Sigma-Aldrich), 50 μM chloroquine (cat. no.: C6628, Sigma-Aldrich), 10 μg/mL cycloheximide (cat. no.: 01810, Sigma-Aldrich), 1 μM MLN4924 (cat. no.: 505477, Calbiochem, San Diego, CA, USA), 5 μM GF109203X (cat. no.: S7208, Selleckchem, Houston, TX, USA), 5 μM AZD1208 (cat. no.: S7104, Selleckchem), 5 μM AZD7762 (cat. no.: S1532, Selleckchem), 5 μM LY2606368 (cat. no.: S6385, Selleckchem), and 300 nM Taxotere (docetaxel trihydrate, Sanofi-Aventis Deutschland GmbH, Frankfurt, Germany), as indicated.

### Stable gene transfer for gene silencing and overexpression

To establish stable knockdown cells through gene silencing via lentiviral transduction, HEK293T cells were transfected with pLKO.1-sh-vector constructs containing short hairpin (sh)-RNAs targeting CUL3 (TRCN0000073343, TRCN0000073344, TRCN0000073345, TRCN0000073346, and TRCN0000073347), SPOP (TRCN0000122224, TRCN0000139043, TRCN0000139181, and TRCN0000145024), ELK3 (TRCN0000013881), CHK1 (TRCN0000000502 and TRCN0000039855), or CHK2 (TRCN0000009947, TRCN0000010209, and TRCN0000399945), along with packaging plasmids (pMD2.G and psPAX2). To establish stable overexpression cells via lentiviral transduction, HEK293T cells were transfected with pCDH-CMV vectors containing the open reading frame for SPOP-wt or SPOP mutants (Y87C, F102C, W131G, and F133V), along with packaging plasmids. After 48 h of cell culture, the viral particles in the cell culture medium were harvested and filtered through a 0.45-μm syringe filter. The harvested virus-containing culture medium was used to infect the indicated cancer cells for gene silencing or overexpression, in the presence of 4 μg/mL polybrene (Thermo Fisher Scientific). Non-viral infected cells were selected by treating them with puromycin (2 μg/mL, Thermo Fisher Scientific) for two days.

### Real-time PCR

Hela cells stably expressing sh-mock or sh-SPOP were seeded into 60-mm dishes and cultured overnight. Total RNA was extracted from the cells using TRIzol (Invitrogen, Carlsbad, CA). The expression of ELK3 gene was analyzed using its specific real-time primer set (Hs00987814_m1) and TaqMan RNA-to-CT 1-step kit (Applied Biosystems) following the manufacturer’s protocol. Relative ELK3 gene expression was normalized against glyceraldehyde 3-phosphate dehydrogenase (GAPDH).

### In vitro kinase assay

The open reading frame of ELK3-wt and ELK3-ΔDeg1 were subcloned into the pET-28a-His vector and expressed in rosetta BL21 *E. coli*. His-tagged-ELK3-wt and ELK3-ΔDeg1 were partially purified through Ni-NTA bead pulldown followed by elution with imidazole. Subsequently, the partially purified ELK3-wt (1 μg) and ELK3-ΔDeg1 (1 μg) were combined with active CHK1 (cat. no.: C47-10H, SignalChem, Richmond, BC, Canada) and CHK2 (cat. no.: C48-10G, SignalChem) and cold ATP. The kinase reaction was conducted for 30 min at 30 °C and terminated by adding a 6× sample buffer. Phosphorylated ELK3 was visualized by WB using an RxxS/T phospho-specific primary antibody and an HRP-conjugated secondary antibody.

### In vitro ubiquitination assay

In vitro ubiquitination assay was performed to evaluate the SPOP-mediated ELK3 ubiquitination. HEK293T cells (3 × 10^6^) were seeded into 100-mm culture dishes and incubation overnight, transfected with pCMV-Myc-SPOP. The cells were treated with MG132 4 h, harvested, and lysed. The Myc-SPOP proteins obtained by IP were used for in vitro ubiquitination assay by adding His-ELK3 (350 ng) obtained from *E. coli* as a substrate and UbcH3/Ubc5a obtained from the kit as an E2 enzyme. His-ELK3, Myc-SPOP, E1, E2, and Biotin-Ubi were mixed in accordance with manufacturer’s protocol (Ubiquitinylation kit, Enzo Life Science, NY, USA). Mixture was incubated at 37 °C for 1 h and quenched the assay with 2× non-reducing loading buffer for western blotting.

### Phos-tag gel electrophoresis

To validate the CHK2-mediated ELK3 phosphorylation, the phos-tag gel electrophoresis was performed. HEK293T cells (3 × 10^6^) were seeded into 100 mm culture dishes, cultured overnight. His-ELK3-WT, -ELK3-S133A, and Flag-CHK2 were transfected as indicated, and the chemical inhibitor was treated as noted. After harvest, the cells were lysed with NP-40 lysis buffer. The protein samples were prepared with whole cell lysate (30 μg) and loaded to phos-tag gel (SuperSep^TM^ Phos-tag^TM^, Cat #: 198-17981, Fuji film, Osaka, Japan). After electrophoresis, the gels were soaked with 10 mM EDTA/Transfer buffer by gentle agitation for 20 min, three times. Subsequently, the gels were re-soaked with EDTA-free transfer buffer for 10 min and blotted using by PVDF membrane. The western blot bands were visualized under a ChemiDoc XRS^+^.

### Immunohistochemistry staining and TissueFAXS analysis

The PCa tissues obtained from PCa patients were subjected to immunohistochemistry (IHC), immunohistofluorescence (ICF), and Hematoxylin/Eosin (H&E) staining following standard protocols. The experiments were approved by the Institutional Review Board of Catholic Medical Center, as described in the ethics explanation. IHC staining was performed as described previously [26]. Briefly, for IHC staining, formalin-fixed paraffin-embedded PCa specimens were sectioned into 3-μm paraffin slices. These sections underwent deparaffinization, rehydration, and antigen unmasking. Subsequently, the tissues were incubated with primary antibodies against ELK3 (dilution 1:200; cat. #: NBP2-01264, Novus) or Brd4 (dilution 1:300; cat. #: ab128874, Abcam, Cambridge, UK) at 4 °C overnight. After three washes with 1× PBS, the sections were exposed to biotinylated secondary antibodies for 1 h, followed by incubation with streptavidin-conjugated HRP for 55 s. Following another set of three washes with 1× PBS, ELK3, and Brd4 were visualized using an HRP/DAB detection kit (Abcam; ab64264). Finally, the tissues were counterstained with hematoxylin (cat. #: 1.05175, Sigma-Aldrich) and eosin (cat. #: 1.02439, Sigma-Aldrich). The stained target proteins were analyzed using the TissueFAXS system (TissueGnostics, Vienna, Austria).

### Identification of *SPOP* mutation in a PCa patient

In November 2020, a 74-year-old male, previously diagnosed with early-stage prostatic adenocarcinoma and treated with curative surgery and adjuvant radiotherapy in 2015, presented at the outpatient clinic for further treatment discussion. Remarkably, over the next five years, he remained in complete remission, showing no signs of cancer recurrence. However, in November 2020, pulmonary metastatic nodules were discovered. Initially, these nodules raised strong suspicions of being metastatic colorectal cancer or primary lung carcinoma. Subsequent surgical biopsy, however, confirmed these lung nodules as metastatic PCa. Clinically, the absence of recurrence in the primary prostate site is exceedingly rare in PCa, particularly considering the common occurrence of bone metastasis in this type of cancer. Furthermore, the patient’s prostate-specific antigen levels remained within the normal range, and distant metastasis was directly observed in the lung, without evidence of bone or lymphatic metastasis. To gain insight into the molecular genetic features of the cancer, including actionable mutations and genetic alterations, the cancer tissue underwent Foundation Medicine’s Next Generation Sequencing (NGS) testing. The NGS results revealed the following information: Microsatellite Status: MS-Stable; Tumor Mutational Burden: 0 Muts/Mb; FANCG splice site: 307 + 1 G > C; and SPOP-F133V mutation.

### Protein–protein docking modeling

The ELK3–SPOP docking modeling was conducted using the Discovery Studio version 2021 computer program (BIOVIA, Inc., San Diego, CA, USA). The 3D structure of SPOP (protein reference database [PDB] ID: 3HQI) was obtained from the Protein Data Bank (https://www.rcsb.org/). The 3D structure of the ELK3 protein was built by the SWISS-MODEL (https://swissmodel.expasy.org/) based on the ELK4 structure. Based on the results of the interaction domain determination between SPOP and ELK3, the SPOP and ELK3 protein structures were subjected to rigid-body simulation of docking poses (ZDOCK) to search all the possible binding models between the two proteins. Approximately 3000 prediction models from ZDOCK were fed into RDOCK to refine and obtain accurate predictions using CHARMm energy minimization. To obtain a reliable complex structure in the solvated system, the best prediction from RDOCK was simulated with water molecules and optional counterions to simulate the solvent, and the solvation model was calculated in Discovery Studio version 2021. The solvated system could be optionally minimized to eliminate van Der Waals clashes. Finally, the interaction between the MATH domain of SPOP and the degron motifs of the serum response factor interaction domain found in ELK3 was successfully emulated using the complementarity-determining regions of the rational antibody–antigen complex model. The rational antibody–antigen complex model satisfied the complementarity-determining regions of the MATH domain of SPOP interacting with the degron motifs of ELK3 at the serum response factor interaction domain. Considering both the ZRANK and RDOCK scores, the top poses were finally selected and subjected to Discovery Studio’s analysis of the binding interface program.

### Ethics approval and consent for participation

The PCa tissues obtained from patients who visited the outpatient department at the Division of Urology, between January 2014 and May 2020, at Seoul and Bucheon St. Mary’s Hospital, were used for IHC, immunofluorescence, and hematoxylin & eosin staining. The study protocol was approved by the Institutional Review Board of the Catholic Medical Center [approval number: HC20SISI0073]. All patients gave their written consent, and this study was performed in accordance with the Declaration of Helsinki II.

### Statistics

All data were derived from three independent experiments rather than technical replicates. Most results are presented as the mean ± SD. Each *n* value is stated in the corresponding figure legend. Statistical significance was calculated using the two-tailed Student’s *t* test or a two-way ANOVA test, as described in the corresponding figure legend. *P*-values less than 0.05 were considered statistically significant (*p < 0.05, **p < 0.01, ***p < 0.001).

## Results

### ELK3 protein is degraded by the CUL3-mediated proteasome pathway

**Fig. 1:**
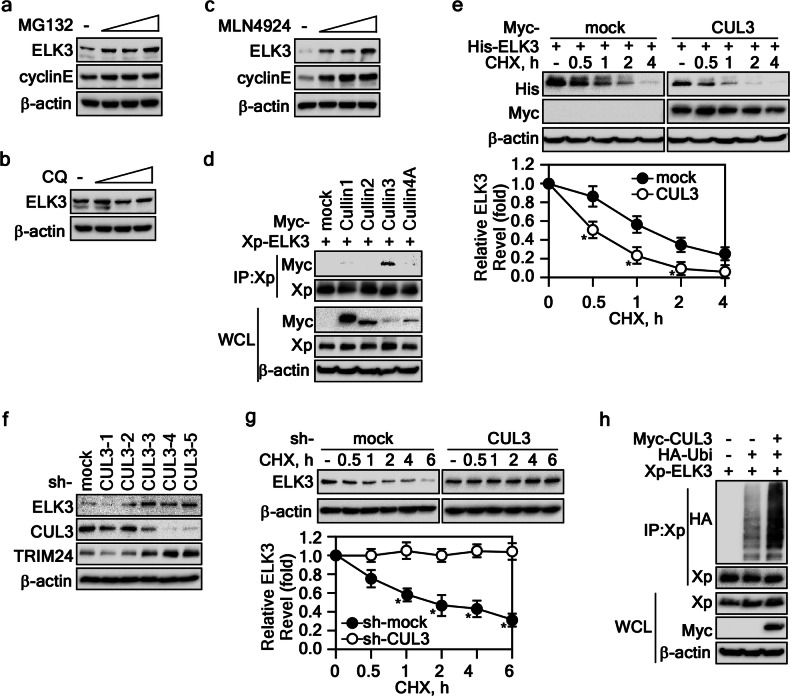
ELK3 stability was regulated by Cul3-mediated proteasome pathway. **a**–**c** Determination of ELK3 degradation pathways. The cell lysates extracted from HeLa cells treated with MG132 (**a**), chloroquine (**b**), or MLN4924 (**c**) were used to visualize ELK3 by WB. **d** ELK3 interaction with Cullin3. The cell lysates extracted from HEK293T cells transfected with indicated plasmids and treated with MG132 (10 μM) for 4 h were used to evaluate the interaction between ELK3 and each of Cullins by IP and WB. **e** Cullin3 decreases ELK3 half-life. The cell lysates extracted from HEK293T cells transfected with indicated plasmids and treated with cycloheximide (CHX, 10 μg/ml) were used to evaluate the half-life of ELK3 by WB. **f** Knockdown of Cullin3 increases ELK3. The cell lysates extracted from HeLa cells stably expressing sh-mock or sh-CUL3 were used to evaluate the ELK3 protein levels by WB. **g** Cullin3 knockdown inhibits ELK3 destabilization. The cell lysates extracted from PC-3 cells stably expressing sh-mock or sh-Cul3 and treated with CHX (10 μg/ml) were used to evaluate the ELK3 half-life by WB. **h** Cullin3 induces ELK3 ubiquitination. The cell lysates extracted from HEK293T cells transfected with indicated plasmids and treated with MG132 (10 μM) for 4 h were used to evaluate the ELK3 ubiquitination by IP and WB. Graphs in **e** and **g** The band intensity ELK3 bands measured using NIH image J computer program and normalized by β-actin intensity were plotted. Data, three independent experiments; Significance, *p < 0.05 obtained by Student *t* test.

Our prior investigations have firmly established the central role of the RSK2-ELK3 signaling pathway in cellular transformation [13]. Furthermore, a thorough examination of the amino acid sequence of ELK3 unveiled a putative SPOP degron motif, specifically encompassing residues 129 to 133 (LRSTS), which align with a Φ (nonpolar) Π (polar) S/T S/T S/T pattern localized within the linker and B domains [13]. To unravel the intricate degradation pathways that oversee the modulation of ELK3 protein stability, we adopted chemical inhibitors, specifically MG132 targeting the proteasomal pathway and chloroquine targeting the lysosomal pathway. These inhibitors were administered to cells to investigate their influence on ELK3 protein levels. Our experimental results compellingly indicated that MG132 treatment significantly elevated the ELK3 protein level (Fig. 1a), whereas chloroquine had no discernible effect (Fig. 1b). This outcome strongly substantiates that the primary mechanism governing ELK3 protein stability is proteasomal degradation. Building upon our earlier hypothesis suggesting SPOP’s potential role as an E3 ligase for ELK3, we conducted experiments employing MLN4924, a well-established inhibitor of Cullin activity. Our results clearly demonstrated that MLN4924 effectively restored ELK3 protein levels in a dose-dependent manner in HeLa cells (Fig. 1c). Furthermore, in-depth explorations into the interaction patterns between ELK3 and members of the Cullin family revealed a specific and selective interaction between ELK3 and CUL3, with no observed interactions with CUL 1, 2, or 4 A (Fig. 1d). This specific interaction strongly supports the premise that ELK3 actively participates in the assembly of a Cullin-RING-based BCR (BTB-CUL3-RBX1) E3 ligase complex. Notably, our investigations into ELK3 turnover dynamics unveiled that co-expression of ELK3 and SPOP significantly accelerated the reduction in ELK3 levels and the shortening of ELK3’s half-life (Fig. 1e). Conversely, genetic depletion of CUL3 through the use of sh-RNA resulted in elevated ELK3 protein levels and an extension of ELK3 protein half-life (Fig. 1f, g). Most strikingly, we observed a substantial enhancement in ELK3 ubiquitination when cells co-expressed CUL3 and ELK3 (Fig. 1h). Collectively, these findings provide compelling evidence that CUL3 serves as an essential adaptor protein, facilitating the formation of a BTB-CUL3-RBX1-ELK3 complex that drives ELK3 ubiquitination, ultimately culminating in its degradation.

### SPOP targets ELK3 ubiquitination and destruction

**Fig. 2:**
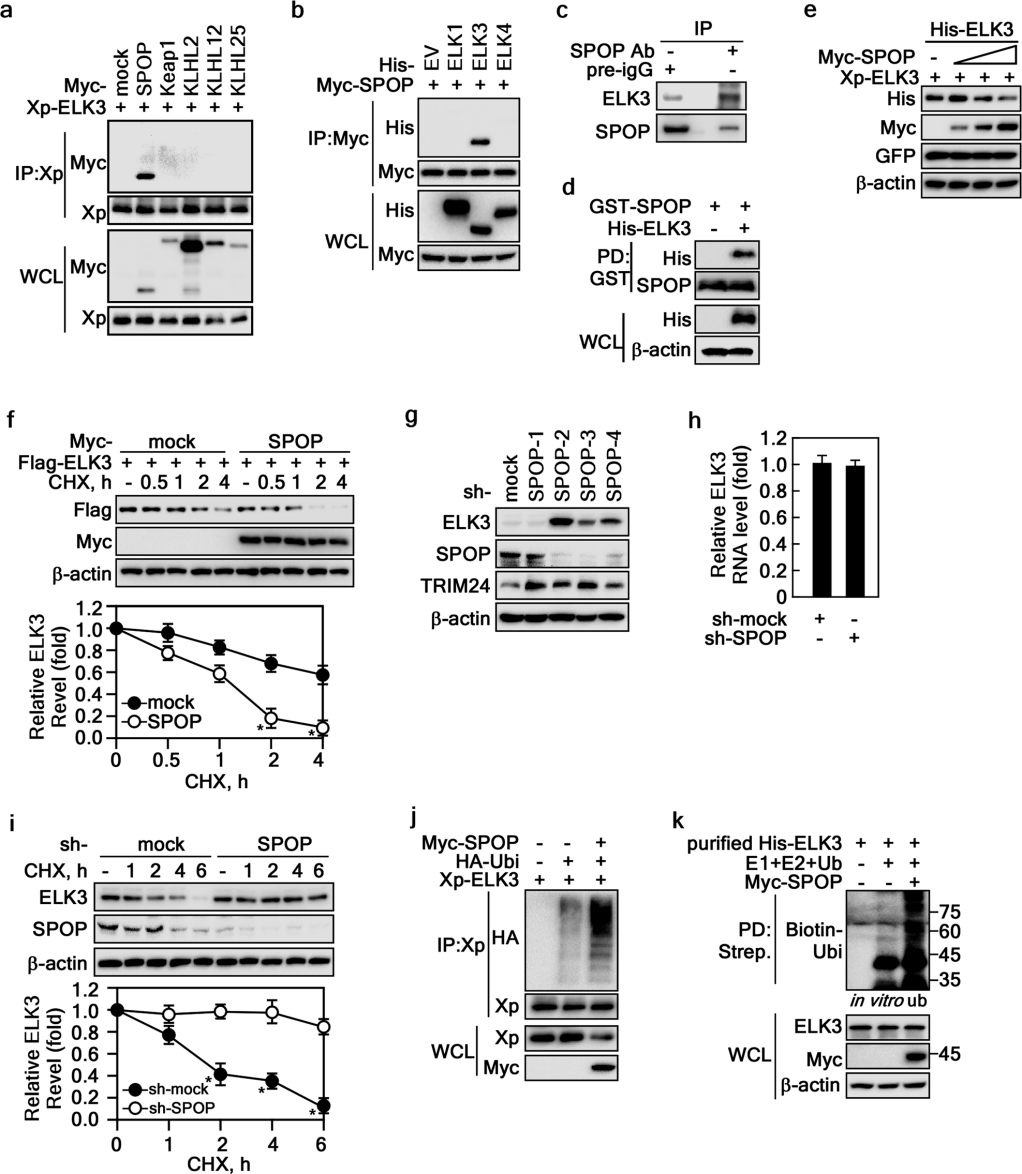
SPOP interacts with ELK3 for its ubiquitination and degradation. **a**, **b** ELK3 interacts with SPOP. The cell lysates extracted from HEK293T cells transiently transfected with indicated plasmids and treated with MG132 (10 μM) for 4 h were used to determine ELK3 interaction to E3 ligases by IP and WB. **c** Evaluation of endogenous interaction between ELK3 and SPOP. The cell lysates extracted from HeLa cells treated with MG132 (10 μM) for 8 h were used to evaluate the ELK3 and SPOP interaction by IP and WB. **d** Confirmation of ELK3 and SPOP interaction. The cell lysates extracted from HEK293T cells transiently transfected with His-ELK3 plasmid were used to confirm the ELK3 and partially purified bacterial His-SPOP by pulldown assay (PD) and WB. **e** SPOP induces ELK3 destabilization. The cell lysates extracted from HEK293T cells transiently transfected with His-ELK3 and dose-increasing Myc-SPOP plasmids were used to evaluate ELK3 protein level by WB. GFP was used as an internal control for equal transfection. **f** SPOP accelerates ELK3 destabilization. The cell lysates extracted from HEK293T cells transiently transfected with indicated plasmids and treated with CHX (10 μg/ml) were used to evaluate the ELK3 half-life by WB. **g** SPOP knockdown increases ELK3 protein levels. The cell lysates extracted from HeLa cells stably expressing sh-mock or sh-SPOP were used to evaluate the ELK3 protein levels by WB. **h** Depletion of SPOP did not affect ELK3 RNA expression. The levels of ELK3 mRNA extracted from HeLa cells stably expressing sh-mock or sh-SPOP were assessed by quantitative real-time RT-PCR using TaqMan RNA-to-CT 1-step kit. **i** SPOP knockdown prolongs ELK3 half-life. The cell lysates extracted from PC-3 cells stably expressing sh-mock or sh-SPOP and treated with CHX (10 μg/ml) were used to evaluate ELK3 half-life by WB. **j**, **k** SPOP induces ELK3 ubiquitination in cell and in vitro ubiquitination assay system. The cell lysates extracted from HEK293T cells transiently transfected with indicated plasmids and treated with MG132 (10 μM) for 4 h were used to evaluate ELK3 ubiquitination by IP and WB (**j**). In vitro ubiquitination assay was conducted using commercially available E1, E2, and ubiquitin (Ubi) proteins, along with His-tagged ELK3 purified from *E. coli* and Myc-tagged SPOP isolated from HEK293T cells overexpressing SPOP (**k**). Graphs in **f** and **i** The band intensity of ELK3 quantified by NIH image J computer program normalized by β-actin intensity was plotted. Data, three independent experiments; Significance, *p < 0.05 obtained by Student *t* test.

The CUL3-based E3 ligase complex orchestrates the recruitment of its substrates through BTB-containing adaptor proteins, which include Kelch-like ECH-associated protein 1 (Keap1), Kelch-like proteins (KLHLs), and SPOP [27]. To elucidate the specific E3 ligase responsible for the interaction with ELK3, we performed immunoprecipitation (IP) experiments. Employing an Xpress antibody for IP and a Myc antibody for subsequent Western blotting, our analysis clearly revealed that ELK3 interacted specifically with SPOP, but not with Keap1, KLHL2, KLHL12, or KLHL25 (Fig. 2a). Furthermore, our investigations indicated that SPOP exhibited an exclusive interaction with ELK3, with no observable interactions with ELK1 or ELK4 (Fig. 2b). This observation confirms that the interaction between ELK3 and SPOP is both specific and selective. Importantly, we validated this interaction not only in endogenous IP (Fig. 2c) but also through an ex vivo pulldown assay utilizing bacterially expressed GST-SPOP (Fig. 2d). These findings collectively establish ELK3 as a genuine binding partner of SPOP. Notably, when co-expressed with SPOP, ELK3 protein levels exhibited a gradual decrease in a dose-dependent manner (Fig. 2e). Similarly, to CUL3 co-expression, the co-expression of SPOP and ELK3 expedited the shortening of ELK3’s half-life compared to mock control cells (Fig. 2f). Conversely, the depletion of SPOP via sh-RNA-SPOP led to increased protein levels of both ELK3 and TRIM24, a known substrate of SPOP (Fig. 2g). This effect was not mirrored at the RNA expression level, as ELK3 mRNA levels remained unchanged (Fig. 2h). Moreover, the ELK3 protein half-life was notably extended in SPOP knockdown Du145 prostate cancer cells (Fig. 2i). Finally, our ubiquitination assay conclusively demonstrated a substantial increase in ELK3 ubiquitination upon co-expression with SPOP in cell (Fig. 2j) and in vitro ubiquitination system assay (Fig. 2k). These comprehensive results unequivocally establish SPOP as the E3 ligase responsible for catalyzing ELK3 ubiquitination, ultimately resulting in the destabilization and degradation of ELK3.

### PCa-associated SPOP mutations abolish ELK3 destabilization

**Fig. 3:**
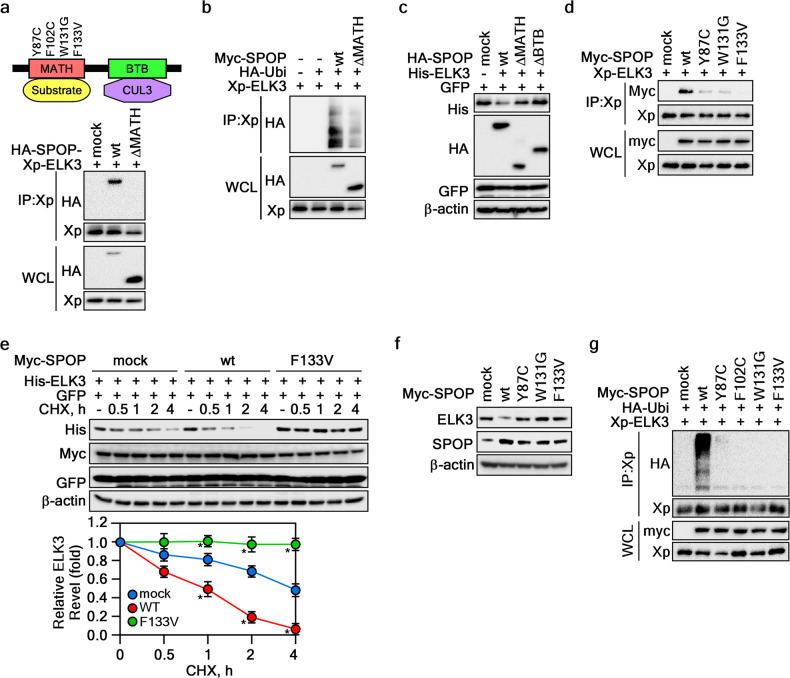
Cancer-derived SPOP mutations fail to promote ELK3 degradation. **a** ELK3 interacted with MATH domain of SPOP. Upper panel Schematic diagram of SPOP domains. The numbers at the MATH domain indicates SPOP mutations observed in PCa patients. Bottom panels The cell lysates of HEK293T cells transiently transfected with indicated plasmids and treated MG132 (10 μM) for 4 h were used to determine the SPOP’s binding domain by IP and WB. **b** MATH domain deletion of SPOP suppressed ELK3 ubiquitination. The cell lysates of HEK293T cells transiently transfected with indicated plasmids and treated MG132 (10 μM) for 4 h were used to evaluate the SPOP-dependent ELK3 ubiquitination by IP and WB. **c** The deletion of the MATH or BTB domain of SPOP abolishes ELK3 destabilization. The cell lysates of HEK293T cells transiently transfected with indicated plasmids were used to evaluate ELK3 protein levels by WB. GFP was used as an internal control for equal transfection. **d** MATH domain mutation of SPOP abrogates interaction with ELK3. The cell lysates of HEK293T cells transiently transfected with the indicated plasmids and treated with MG132 (10 μM) for 4 h were used to evaluate the interaction between SPOP and ELK3 by IP and WB. **e** MATH-F133V mutation of SPOP critically affects ELK3 stability. The cell lysates of HEK293T cells transiently transfected with indicated plasmids and treated with CHX (10 μg/ml) for indicated period were used to evaluate ELK3 protein levels by WB. The band intensity of ELK3 quantified by NIH image J computer program normalized by β-actin intensity was plotted. GFP was used an internal control for equal transfection. Data, three independent experiments; Significance, *p < 0.05 obtained by Student *t* test. **f** SPOP mutations at MATH domain inhibits endogenous ELK3 destabilization. The cell lysates of 22Rv1 cells stably expressing SPOP-wt or each of SPOP mutants (Y87C, W131G, and F133V) were used to evaluate ELK3 protein levels by WB. **g** SPOP mutations at MATH domain abolishes ELK3 ubiquitination. The cell lysates of HEK293T cells transiently transfected with indicated plasmids and treated with MG132 (10 μM) for 4 h were used to evaluate ELK3 ubiquitination by IP and WB.

SPOP’s recognition and ubiquitination of substrates are well-established functions mediated through its MATH domain [14]. Intriguingly, mutations found in human prostate cancer (PCa) patients, specifically Y87C, F102C, W131G, and/or F133V, predominantly cluster within the MATH domain (Fig. 3a). These mutations disrupt substrate binding [22]. To investigate whether SPOP’s interaction with ELK3 follows the same mode of action via the MATH domain, we generated a MATH domain deletion mutant (HA-SPOP-ΔMATH). IP experiments using HA-SPOP-wt or -ΔMATH revealed that deletion of the MATH domain completely abolished ELK3 binding (Fig. 3a) and subsequent ubiquitination (Fig. 3b). Furthermore, deletion of either the MATH or BTB domains increased ELK3 protein levels, suggesting that MATH-mediated ELK3 binding and BTB-mediated CUL3 complex formation is necessary to ELK3 ubiquitination and degradation (Fig. 3c). Crucially, the frequently observed SPOP mutations in the MATH domain in PCa patients, such as Y87C, W131G, or F133V, led to a total loss of interaction with ELK3 (Fig. 3d). Notably, this disrupted interaction with SPOP mutants resulted in sustained ELK3 protein levels during cycloheximide (CHX) treatment, in stark contrast to the time-dependent reduction observed with SPOP-wt (Fig. 3e). Additionally, while SPOP-wt co-expression led to a decrease in ELK3 protein levels, SPOP co-expression with Y87C, W131G, or F133V in DU145 cells maintained ELK3 protein levels (Fig. 3f). In line with these findings, the enhanced ELK3 ubiquitination induced by SPOP-wt was effectively blocked by SPOP mutations at Y87C, F102C, W131G, or F133V within the MATH domain (Fig. 3g). These comprehensive results affirm that SPOP destabilizes ELK3, with the MATH domain of SPOP playing a pivotal role in mediating its interaction with ELK3.

### Determination of the ELK3 degron motif for SPOP

**Fig. 4:**
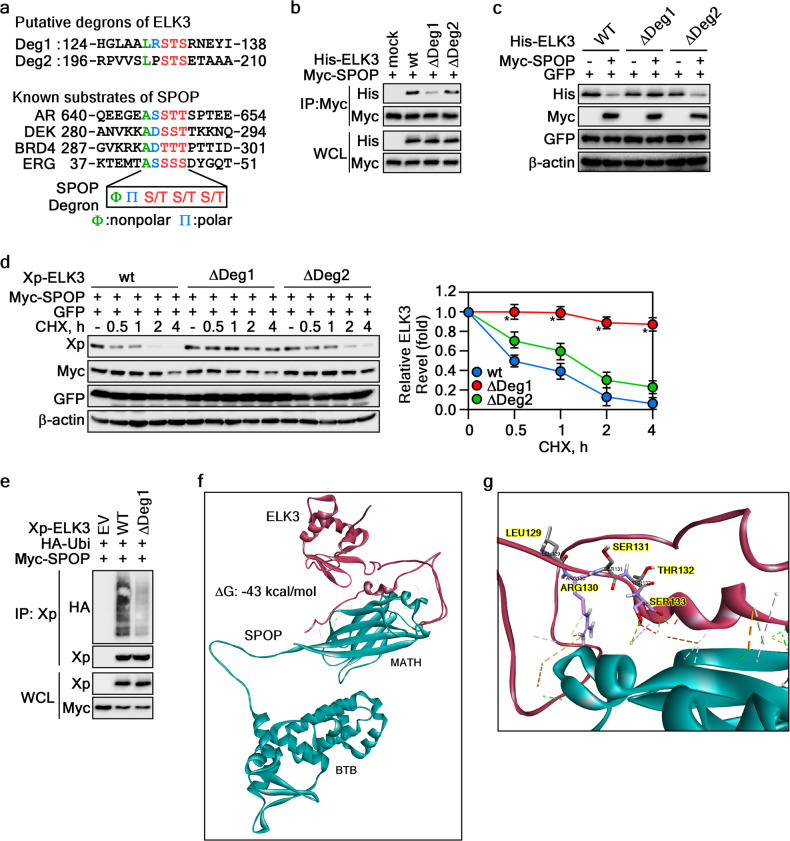
Determination of ELK3 degron motif for SPOP. **a** Identification of ELK3 degron motifs for SPOP. ELK3 amino acid sequences were aligned to search putative degron motifs for SPOP. **b** Determination of ELK3 degron motif interacting to SPOP. The cell lysates of HEK293T cells transiently transfected with indicated plasmids and treated with MG132 (10 μM) for 4 h were used to evaluate the ELK3 degron motif by IP and WB. **c** Involvement of ELK3 Deg1 in its stabilization. The cell lysates of HEK293T cells transiently transfected with indicated plasmids were used to determine the ELK3 degron motif by WB. GFP was used an internal control for equal transfection. **d** ELK3 Deg1 deletion abrogates SPOP-mediated destabilization. Left panels The cell lysates of HEK293T cells transiently transfected with indicated plasmids and treated with CHX (10 μg/ml) for an indicated time were used to evaluate the ELK3 protein levels by WB. GFP was used as an internal control for equal transfection. Graph The band intensity of ELK3 quantified by NIH image J computer program normalized by β-actin intensity was plotted. GFP was used an internal control for equal transfection. Data, three independent experiments; Significance, *p < 0.05 obtained by Student *t* test. **e** ELK3 Deg1 deletion abolishes SPOP-mediated ubiquitination. The cell lysate of HEK293T cells transiently transfected with indicated plasmids and treated with MG132 (10 μM) for 4 h were used to evaluate SPOP-mediated ELK3 ubiquitination by IP and WB. **f**, **g** Protein–protein docking of ELK3 and SPOP. The SPOP structure (PDB ID: 3HQI) and ELK3 built by amino acid similarly modeling using Discovery Studio were used to predict SPOP and ELK3 interaction interface. Magenta, ELK3; Green, SPOP. The amino acids and interaction types involved in interaction between ELK3 and SPOP are provided in Supplementary Fig. S1.

As the recognition of substrate proteins by SPOP hinges on the presence of a consensus degron motif, Ф-П-S/T-S/T-S/T (Ф: nonpolar, П: polar) [14], we conducted a thorough analysis of ELK3’s amino acid sequences to identify potential degron motifs. Our analysis unveiled two potential SPOP binding motifs situated at the juncture of the 1st linker and B domains (amino acid [aa] 129–133, designated as Deg1) and the 2nd linker and NID domains (aa 201–205, designated as Deg2) (Fig. 4a). Intriguingly, while deletion of Deg1 (ΔDeg1) significantly diminished the interaction between SPOP and ELK3 (Fig. 4b), deletion of Deg2 (ΔDeg2) had no such effect, suggesting that the LRSTS motif of ELK3 at aa 129–133 may function as the degron motif recognized by SPOP. Importantly, ΔDeg1 not only abolished SPOP-mediated ELK3 degradation (Fig. 4c) but also prolonged the half-life of ELK3 (Fig. 4d). These findings lend support to our hypothesis that the LRSTS motif of ELK3 at aa 129–134 is crucial for its interaction with SPOP. Notably, ΔDeg1 of ELK3 prevented SPOP-induced ubiquitination, which was robustly observed in ELK3-wt (Fig. 4e). Collectively, these results establish that the Deg1 motif of ELK3 within aa 129–134 serves as a critical degron motif for its interaction with SPOP. However, while these cutting-edge molecular biology approaches have illuminated this interaction, the precise molecular mechanisms governing ELK3 and SPOP interaction have not been fully elucidated. Consequently, we conducted a protein–protein docking analysis using the Discovery Studio computational modeling system. This analysis utilized the 3D protein structures of ELK3 and SPOP (PDB: 3HQI) retrieved from the Protein Data Bank (PDB) (https://www.rcsb.org). The results of the protein–protein interaction docking modeling yielded 3000 binding poses. These models were then sorted based on ZDOCK and RDOCK scores, with 50 potential poses selected for further examination. Among these 50 poses, we manually validated the interaction, focusing on Y87, F102, W131, and F133 of the SPOP-MATH domain and aa 129–134 of ELK3. Ultimately, we identified the optimal model depicting the mode of interaction between ELK3 and SPOP (Fig. 4f). A more detailed analysis of the binding interface between ELK3 and SPOP proteins revealed that this interaction was characterized by 8 hydrophobic interactions, 11 hydrogen bonds, and 6 electrostatic interactions, with a *Δ*G value of –43 kcal/mol (Supplementary Fig. S1). Notably, Arg130 and Ser133 of the ELK3 degron motif emerged as pivotal participants in the binding to SPOP (Fig. 4g and Supplementary Fig. S1). These findings collectively demonstrate that SPOP-mediated ELK3 destabilization hinges on the interaction between the MATH domain of SPOP and the degron motif of ELK3 located at aa 129–133.

### CHK1/2 facilitates ELK3 protein ubiquitination and degradation

**Fig. 5:**
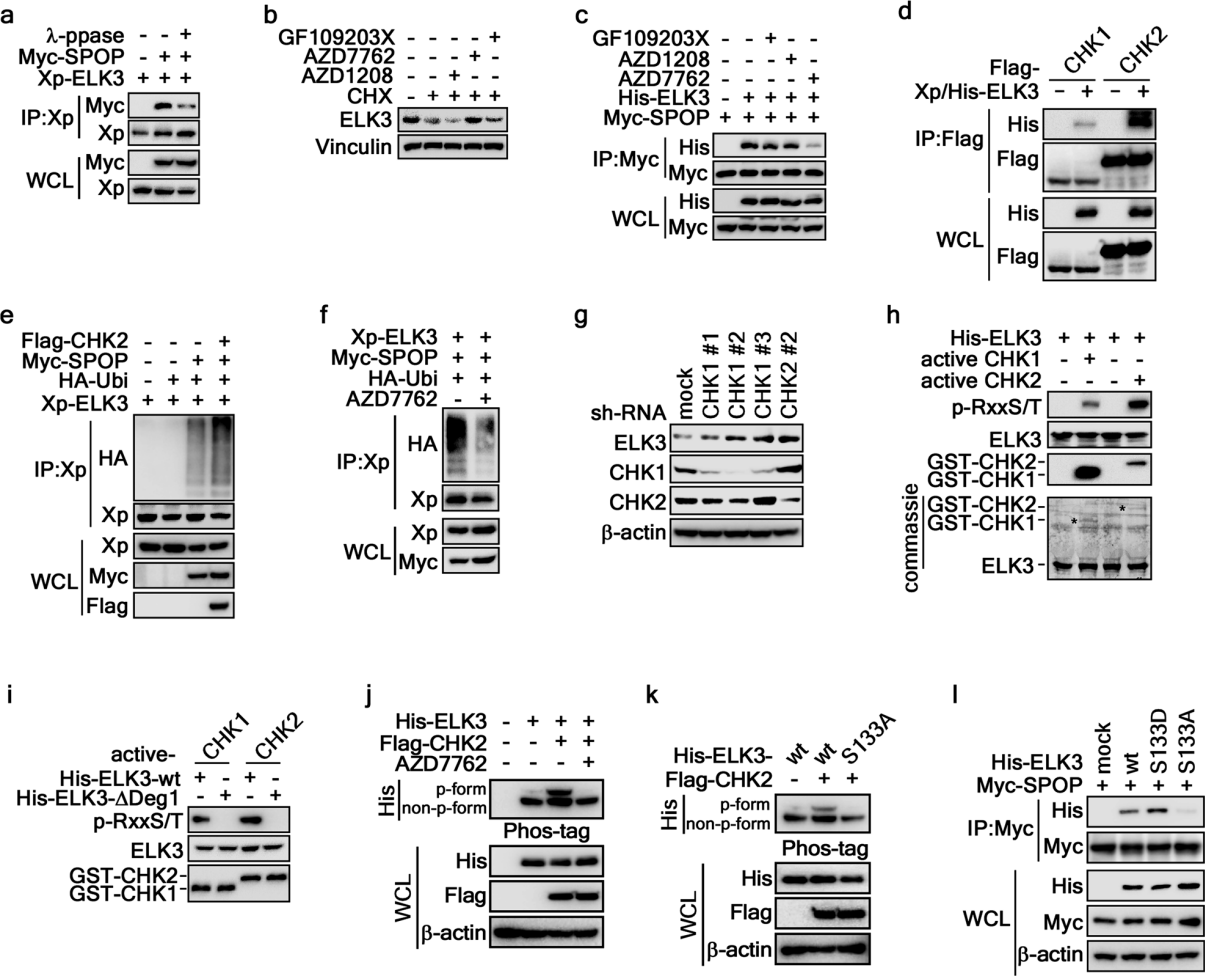
CHK1/2-mediated ELK3 phosphorylation facilitates ELK3 destruction. **a** Dephosphorylation of ELK3 prevent the interaction between ELK3 and SPOP. The cell lysates of HEK293T cells transiently transfected with indicated plasmids were treated with/without λ-phosphatase. The interaction between ELK3 and SPOP was evaluated by IP and WB. **b** CHK1/2 inhibition suppresses ELK3 destabilization. The cell lysates of HeLa cells treated with 5 μM of indicated inhibitor and CHX (10 μg/ml) for 12 h were used to evaluate the ELK3 protein levels by WB. **c** CHK1/2 inhibition abolishes ELK3 and SPOP interaction. The cell lysates of HEK293T cells transfected with indicated plasmids treated with 5 μM of indicated inhibitor for 12 h and MG132 (10 μM) for 4 h were used to evaluate the interaction between ELK3 and SPOP by IP and WB. **d** ELK3 interacts with CHK1 and CHK2. The cell lysates of HEK293T cells transiently transfected with indicated plasmids and treated with MG132 (10 μM) for 4 h were used to evaluate the interaction between ELK3 and CHK1 or CHK2 by IP and WB. **e** CHK2 facilitates ELK3 ubiquitination by SPOP. cell lysates of HEK293T cells transiently transfected with indicated plasmids and treated with MG132 (10 μM) for 4 h were used to evaluate the ELK3 ubiquitination by IP and WB. **f** CHK1/2 inhibition abrogates SPOP-mediated ELK3 ubiquitination. The cell lysates of HEK293T cells transiently transfected with indicated plasmids and treated with 5 μM of AZD7762 for 12 h and MG132 (10 μM) for 4 h were used to evaluate the SPOP-mediated ELK3 ubiquitination by IP and WB. **g** CHK1 knockdown increases ELK3 protein levels. The cell lysates of HeLa cells stably expressing sh-mock or sh-CHK1 or CHK2 were used to evaluate ELK3 protein levels by WB. **h** CHK1 and CHK2 phosphorylates ELK3. In vitro kinase assay using partially purified His-ELK3 and active CHK1 or CHK2 was conducted. ELK3 phosphorylation by CHK1 or CHK2 was evaluated RxxS/T antibody by WB. **i** ELK3 deg1 deletion abolishes CHK1- or CHK2-mediated phosphorylation. In vitro kinase assay using partial purified His-ELK3-wt or -∆Deg1 and active CHK1 or CHK2 was conducted. ELK3 phosphorylation by CHK1 or CHK2 was evaluated RxxS/T antibody by WB. **j**, **k** CHK2 phosphorylates ELK3 at Ser133 in cell system. The phosphorylation of ELK3 mediated by CHK2 in cell culture (**j**) and in vitro kinase assay system (**k**) was evaluated using phos-tag immunoblot analysis. Treatment with AZD7762 (**j**) and mutation of ELK3 Ser133 to Ala (**l**) abolished the phosphorylation of ELK3 induced by CHK2. **l** ELK3 phosphorylation at Ser133 is indispensable to interact with SPOP. ELK3 phosphorylation requirement for the interaction with SPOP was evaluated by IP and western blotting using cell lysates transiently expressing mock, His-ELK3-wt, His-ELK3-S133D, or His-ELK3-S133A.

For the recognition to occur between SPOP and its substrate, phosphorylation of the substrate at the degron motif is an essential prerequisite [18, 19, 24]. In line with this premise, we conducted an IP analysis to investigate the impact of ELK3 dephosphorylation on its interaction with SPOP. We used cell lysates treated either with or without λ-phosphatase. The results clearly demonstrated that treatment with λ-phosphatase led to a significant reduction in the interaction between ELK3 and SPOP (Fig. 5a), highlighting the critical role of ELK3 phosphorylation by known kinase(s) in its destabilization. To identify the potential kinases responsible for ELK3 phosphorylation, we conducted an analysis and prediction using PhosphoNET (http://www.phosphonet.ca/). This prediction suggested that protein kinase C, serine/threonine-protein kinase PIM2, CHK1, and CHK2 could be potential candidates for ELK3 phosphorylation. To validate these predictions, we performed pharmacological assays using kinase inhibitors, including GF109203X, a protein kinase C inhibitor, AZD1208, a serine/threonine-protein kinase PIM2 inhibitor, and AZD7762, a CHK1/2 inhibitor. Notably, our experiments revealed that the inhibition of CHK1 and CHK2 by AZD7762 led to a significant recovery in the ELK3 protein levels (Fig. 5b) and interfered with the interaction between ELK3 and SPOP (Fig. 5c), providing strong evidence that CHK1/2 could be crucial kinases responsible for ELK3 phosphorylation and subsequent degradation. Intriguingly, the degron motif of ELK3, LRSTS, required for SPOP recognition, perfectly matched the conserved CHK1/2 phosphorylation target motif, R/K-x-x-S/T (Supplementary Fig. S2). As expected, IP experiments using cell lysates transiently co-expressing Xp/His-ELK3 with CHK1 or CHK2 demonstrated that both CHK1 and CHK2 interacted with ELK3 (Fig. 5d). Furthermore, ELK3 ubiquitination induced by co-expression of SPOP was significantly enhanced by the co-expression of SPOP and CHK2 (Fig. 5e). However, this enhanced ubiquitination was effectively abolished by AZD7762 treatment (Fig. 5f), providing strong evidence that CHK1/2-mediated phosphorylation of ELK3 at the degron motif is responsible for ELK3 ubiquitination mediated by SPOP. Conversely, the knockdown of CHK1 or CHK2 resulted in the restoration of ELK3 protein levels (Fig. 5g). Additionally, an in vitro kinase assay using ELK3 partially purified from *E. coli* provided direct evidence that ELK3 serves as a substrate for CHK1 and CHK2 (Fig. 5h). However, the Deg1 deletion mutant of ELK3 completely abrogated CHK1- or CHK2-mediated phosphorylation, as observed with ELK3-wt (Fig. 5i). To further validate the phosphorylation of ELK3 mediated by CHK1/2 in cells, we performed a phos-tag gel electrophoresis and immunoblot analysis. We found that the ectopic expression of CHK2 induced ELK3 phosphorylation (Fig. 5j). The CHK2-induced phosphorylation of ELK3 was markedly abolished by AZD7762 treatment (Fig. 5j). Given the CHK1/2 phospho-motif on ELK3 (Supplementary Fig. 2), we generated an ELK3-S133A mutant. Notably, CHK2-mediated ELK3 phosphorylation was largely abolished in the ELK3-S133A mutant compared to ELK3-wt (Fig. 5k). More importantly, phospho-mimic-ELK3-S133D showed the dramatic increase of SPOP interaction rather than that of ELK3-wt (Fig. 5l). Additionally, ELK3-S113A was totally abolished the interaction with SPOP (Fig. 5l). These results collectively establish that CHK1- and CHK2-mediated phosphorylation of ELK3 enhances SPOP-mediated ELK3 destabilization.

### SPOP-mediated ELK3 degradation inhibits *c-fos* expression and PCa cell migration

**Fig. 6:**
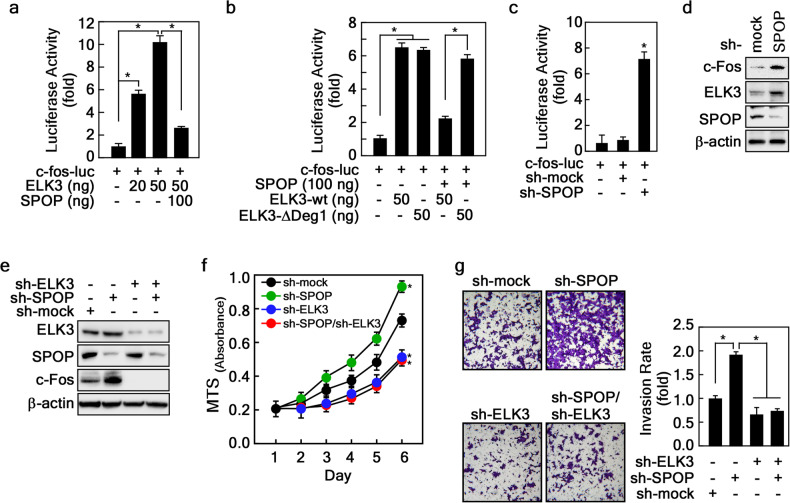
SPOP-mediated ELK3 degradation suppresses *c-fos* expression. **a**, **b** SPOP inhibits ELK3-mediated *c-fos* promoter activity. HEK293T cells transiently transfected with indicated plasmids were used to evaluate *c-fos* promoter activity by luciferase assay. **c** SPOP knockdown increases *c-fos* promoter activity. HEK293T cells stably expressing sh-SPOP were transfected with *c-fos*-luc reporter plasmid and evaluated *c-fos* promoter activity by luciferase assay. **d** Confirmation of increased c-Fos protein by SPOP knockdown. The cell lysates of 22Rv1 cells stably expressing sh-SPOP were used to evaluate the c-Fos protein level by WB. **e** Establishment of ELK3 and SPOP double knockdown cells. The cell lysates of 22Rv1 cells stably expressing sh-SPOP and/or sh-ELK3 as indicated were used to evaluate the SPOP and c-Fos protein levels by WB. **f** Increased cell proliferation by SPOP knockdown suppresses cell proliferation by ELK3. The 22Rv1 cells in **e** were used to evaluate the effects of cell proliferation by CCK-8 assay. **g** Increased cell invasion by SPOP knockdown abrogated by ELK3 knockdown. The 22Rv1 cells in **e** were used to evaluate the effects of cell invasion by cell invasion assay using Matrigel Invasion Chamber. **a**–**c** and **f**, **g** Data, three independent experiments; Significance, *p < 0.05 obtained by Student *t*-test.

Our previous research has elucidated that the induction of *c-fos* expression by ELK3 plays a pivotal role in cell transformation and cancer cell proliferation [13]. As c-Fos is a proto-oncogene implicated in the proliferation, migration, and invasion of various human cancer types [28], we delved into the impact of the SPOP-ELK3 axis on *c-fos* gene expression. To investigate this, we conducted a luciferase reporter assay using the *c-fos* promoter and observed that ELK3 overexpression led to an increase in *c-fos* promoter activity. Conversely, when SPOP and ELK3 were co-expressed, *c-fos* promoter activity was significantly inhibited (Fig. 6a). Furthermore, consistent with our previous findings that ELK3-ΔDeg1 exhibited resistance to SPOP-mediated destabilization, we discovered that ELK3-ΔDeg1 promoted *c-fos* promoter activity independently of SPOP overexpression (Fig. 6b). Additionally, SPOP depletion resulted in an enhancement of *c-fos* promoter activity (Fig. 6c), leading to an increase in ELK3 levels, subsequently elevating c-Fos protein levels in 22Rv1 cells (Fig. 6d). To gain insight into the role of the SPOP-ELK3-c-Fos axis in the proliferation and migration of PCa cells, we established stable cell lines with knockdown of SPOP and/or ELK3 (Fig. 6e). In these cell lines, we confirmed that depletion of ELK3 inhibited the proliferation and invasion of 22Rv1 cells, while SPOP depletion had the opposite effect, enhancing proliferation and invasion in 22Rv1 cells (Fig. 6f, g) and Du145 cells (Supplementary Fig. S3a, b). These findings suggest that SPOP-mediated degradation of ELK3 plays a pivotal role in governing the progression of PCa through the regulation of *c-fos* expression.

### Regulation of ELK3 protein stability by SPOP contributes to docetaxel-induced cell death

**Fig. 7:**
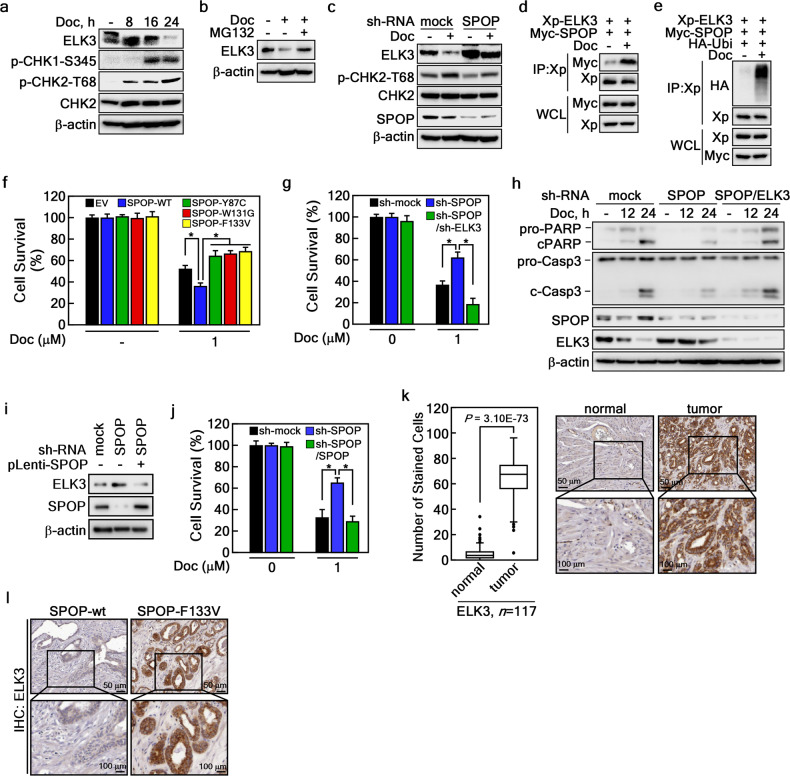
SPOP-ELK3 axis regulates docetaxel-induced cell death. **a** Inverse correlation of ELK3 and phospho-CHK1/2 protein levels under docetaxel treatment. The cell lysates of 22Rv1 cells treated with docetaxel (300 nM) for an indicated time were used to evaluate the indicated protein levels by WB. **b** Rescue of docetaxel-mediated decreased ELK3 protein level by MG132 treatment. The 22Rv1 cell lysates treated with docetaxel (300 nM) in the presence or absence of MG132 (10 μM) were used to evaluate ELK3 protein levels by WB. **c** Docetaxel-mediated decreased ELK3 protein is rescued by SPOP knockdown. The cell lysates of 22Rv1 cells stably expressing sh-SPOP and treated with docetaxel (300 nM) were used to evaluate ELK3 protein level by WB. **d** Docetaxel induces SPOP and ELK3 interaction. The cell lysates of HEK293T cells transfected with indicated plasmids and treated with docetaxel (300 nM) were used to evaluate the SPOP and ELK3 interaction by IP and WB. **e** Docetaxel induces ELK3 ubiquitination. The cell lysates of HEK293T cells transfected with indicated plasmids and treated with docetaxel (300 nM) were used to evaluate ELK3 ubiquitination by IP and WB. **f** Diminished interaction between SPOP and ELK3 increases PCa cell survivability. 22Rv1 cells stably expressing SPOP-WT or each of SPOP mutants treated with docetaxel (1 μM) for 48 h were used to cell survivability by CCK-8 assay. **g** SPOP and ELK3 double knockdown inhibits cell survivability by docetaxel in SPOP knockdown cells. 22Rv1 cells stably expressing sh-SPOP and/or sh-ELK3 treated with docetaxel (1 μM) for 48 h were used to evaluate cell survivability by CCK-8 assay. **h** SPOP and ELK3 double knockdown enhances apoptosis signaling by docetaxel. The cell lysates of 22Rv1 cells stably expressing sh-SPOP and/or sh-ELK3 treated with docetaxel (300 nM) were used to evaluate the protein levels of apoptosis-related signaling molecules by WB. **i**, **j** The SPOP-ELK3 axis plays a critical role in docetaxel-induced cell death. The cell lysates of 22Rv1 cells stably expressing sh-SPOP and/or SPOP were used to evaluate the protein levels of ELK3 and SPOP by WB (**i**), and to assess cell viability using the CCK-8 assay (**j**). **k** Increased ELK3 protein levels in PCa cancer tissues. IHC images of ELK3 in prostate cancer tissues (*n* = 117) stained by DAB staining were analyzed by TissueFAXS system. Data, DAB intensity in normal vs. cancer tissues; Significance was obtained by Anova *t* test. **l** Increased ELK3 protein levels in PCa patient harboring SPOP-F133V mutation. Representative IHC image of ELK3 protein levels by DAB staining in prostate cancer tissues harboring SPOP-WT or SPOP-F133V. **f**, **g** Data, three independent experiments; Significance, *p < 0.05 obtained by Student *t* test.

Accumulating evidence suggests that ELK3 is implicated in the development of anticancer drug resistance, such as resistance to doxorubicin and oxaliplatin treatment in breast and colorectal cancer, respectively [29, 30]. This observation prompted us to hypothesize that the SPOP-ELK3 axis might play a crucial role in anticancer drug resistance in PCa. Initially, we conducted an IC_50_ evaluation for docetaxel on PCa cells, including 22Rv1, DU145, and PC-3. We used a range of docetaxel concentrations from 1 to 3000 nM and assessed cell viability 24 h after treatment using the CCK-8 assay. The determined IC_50_ values were 831.3 nM for 22Rv1, 842.7 nM for DU145, and 1358.0 nM for PC-3 cells (Supplementary Fig. S4). Based on these findings, we selected different docetaxel dosages for subsequent experiments: a low dose of docetaxel (300 nM) was used to assess ELK3 protein stability regulation and the interaction between ELK3 and SPOP, whereas a higher dose of docetaxel (1 μM) was utilized for cell survival analysis. To examine this hypothesis, we treated 22Rv1 cells with docetaxel, a common chemotherapy drug for hormone-refractory PCa. Intriguingly, docetaxel led to a suppression of ELK3 protein levels, while the phosphorylation levels of CHK1 and CHK2 were conversely elevated, suggesting that docetaxel may influence ELK3 protein turnover (Fig. 7a). In order to investigate whether docetaxel is associated with ELK3 protein degradation, we discovered that the reduced ELK3 protein levels caused by docetaxel treatment could be restored by MG132 treatment (Fig. 7b). Building on our previous findings that phosphorylation of ELK3 by CHK1/2 enhances SPOP binding to ELK3 and promotes ELK3 degradation (Fig. 5), we treated SPOP knockdown cells with docetaxel. Remarkably, unlike the sh-mock cells, the ELK3 protein levels did not decrease with docetaxel treatment in sh-SPOP stable cells (Fig. 7c). Moreover, IP results demonstrated that docetaxel induced an interaction between ELK3 and SPOP, leading to increased ELK3 ubiquitination (Fig. 7d, e). These results indicate that docetaxel negatively regulates ELK3 protein stability. Next, we examined how the SPOP-ELK3 axis is involved in docetaxel resistance in PCa cells. 22Rv1 cells stably expressing SPOP mutants, such as SPOP-Y87C, -W131G, or -F133V, exhibited docetaxel resistance compared to mock and SPOP-wt overexpressing cells (Fig. 7f). Furthermore, 22Rv1 cells stably expressing sh-SPOP or sh-SPOP/sh-ELK3 showed a completely opposite response to docetaxel treatment (Fig. 7g). Mechanistically, SPOP depletion suppressed the cleavage of poly-ADP ribose polymerase (PARP) and caspase-3 induced by docetaxel compared to sh-mock control cells (Fig. 7h). Importantly, ELK3 knockdown in sh-SPOP cells restored the cleavage of PARP and caspase-3 upon docetaxel treatment (Fig. 7h), demonstrating that SPOP mutations confer resistance to docetaxel by increasing ELK3 protein stability. To further confirm the effect of SPOP-mediated ELK3 degradation on docetaxel-induced cell death, we reintroduced SPOP into SPOP-depleted cells using lentiviral system. The ELK3 protein level, increased by SPOP knockdown, was suppressed by the re-introduction of SPOP (Fig. 7i). Consistently, reintroducing SPOP into sh-SPOP cells re-sensitized docetaxel-induced cell death, as shown as sh-mock cells (Fig. 7j). The clinical relevance of ELK3 protein abundance in PCa tissues was confirmed by an IHC assay using an ELK3-specific antibody in PCa patients (*n* = 117, Supplementary Fig. S5). IHC analysis revealed that ELK3 protein levels were significantly higher in tumor tissues compared to the normal tissues surrounding tumors (Fig. 7k). Parallel IHC with a specific antibody for BRD4, a well-known SPOP substrate, displayed similar increased patterns in tumor tissues (Supplementary Fig. S6a). Interestingly, among the 117 tumor tissues, we identified one sample with an SPOP mutation at F133V by NGS. Notably, ELK3 protein levels were markedly elevated in the SPOP-mutant tumor tissue compared to the tissue surrounding the normal area (Fig. 7l). Additionally, the protein level of BRD4 was increased in the same specimen (Supplementary Fig. S6b). These findings collectively demonstrate that SPOP mutations confer docetaxel resistance by increasing ELK3 protein stability.

## Discussion

Androgen deprivation therapy has long been a standard treatment for prostate cancer (PCa), initially showing effectiveness. However, a significant challenge arises when PCa progresses to the life-threatening stage of castration-resistant prostate cancer (CRPC) [3]. In an attempt to address this, docetaxel/prednisone was approved by the FDA in 2004 as a treatment option [4]. While docetaxel has shown promise in extending patient survival, resistance often develops over time, rendering it less effective [8]. The mechanisms underlying docetaxel resistance in PCa are complex and multifaceted. Some factors contributing to resistance include mutations in βI-tubulin, specifically the βI-tubulin-F270I mutation, which affects the docetaxel binding site, as well as the overexpression of TUBB3 [31, 32]. Additionally, the presence of androgen receptor (AR) variants, such as AR-v7 and AR-v567es, which remain active in the absence of dihydrotestosterone, contribute to PCa progression by driving AR target gene transcription [33]. Beyond these known causes, there are several other suggested mechanisms of docetaxel resistance in PCa. These include ETS-related gene rearrangements, which interact directly with β-tubulin [34], the induction of p-glycoprotein/ATP-binding cassette subfamily B member 1 to mediate docetaxel efflux [35], decreased docetaxel influx due to SLCO1B3 suppression [36], and the induction of cancer stem cell populations marked by CD44, CD133, and ALDH expression [37]. However, there remains a scarcity of studies exploring the molecular mechanisms of docetaxel resistance in PCa comprehensively.

**Fig. 8:**
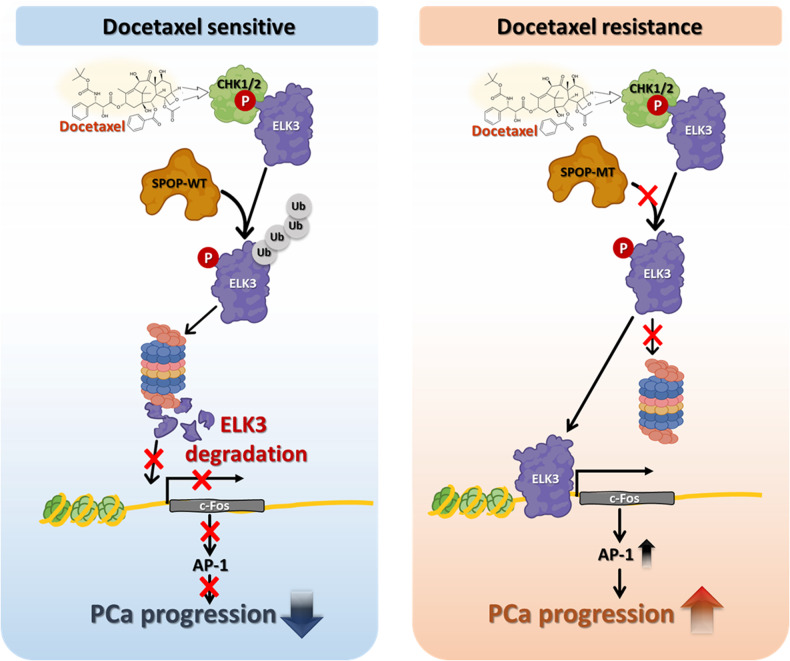
Graphical summary of the proposed mechanism. A schematic diagram illustrates the working model in which SPOP mutants enhance ELK3 stabilization, leading to increased resistance to docetaxel-induced cell death. Consequently, the SPOP-ELK3 axis plays a pivotal role in the progression of prostate cancer (PCa).

In this context, the role of ELK3 in PCa malignancy and metastasis is of particular interest. Previous research has suggested that targeting the Ras/ERK-mediated activation of ELK3 with XRP44X, an inhibitor, can suppress tumor growth and metastasis in mice [38]. Furthermore, studies have shown that silencing ELK3 using siRNA leads to increased apoptosis and decreased cell proliferation and migration in Du145 PCa cells [12]. Our research group previously found that the RSK2-ELK3 signaling pathway plays a key role in cell transformation and cancer cell proliferation via *c-fos* gene expression [13]. It highlights that ELK3’s impact on cancer cell proliferation and malignancy is not only mediated through phosphorylation but also through its regulation of protein stability. Specifically, the study reveals that the E3 ubiquitin ligase SPOP plays a crucial role in regulating ELK3 stability. While previous research has indicated that hypoxia induces ELK3 ubiquitination and degradation through the proteasome [39], the precise mechanisms and implications of this process in cancer development have yet to be fully elucidated. The findings presented here suggest that targeting ELK3 activation and its stability-enhancing pathways may hold promise as therapeutic strategies for addressing docetaxel resistance in PCa (Fig. 8). This research sheds light on a potential avenue for improving the treatment of CRPc and warrants further investigation.

Although c-FOS is not exclusively a substrate of ELK3, it is indeed correct that ELK3, also known as Net (ERP/SAP-2), functions as a transcription factor that is crucial for the regulation of *c-fos* gene expression through its binding to the SRE within the *c-fos* promoter (*c-fos* SRE) [40]. Our studies further demonstrate that *c-fos* promoter activity is enhanced by ELK3 in a dose-dependent manner [13]. Significantly, ELK3’s activity is pivotal in the proliferation of triple-negative breast cancer cells [13] and androgen-independent human prostate cancers [41]. Moreover, c-FOS, as a part of the AP-1 complex, has been identified with a high frequency across the genome by Chip-Seq analysis [42]. Given its classification as an immediate-early response gene, establishing a direct gene expression profile mediated by ELK3 through ELK3-mediated RNA-seq might present challenges. Consequently, we posited that examining *c-fos* promoter activity could serve as an effective strategy to assess SPOP-mediated regulation of ELK3 activity (Fig. 6).

In the present study, we identified CUL3/SPOP as an E3 ligase for ELK3 ubiquitination. SPOP specifically interacted with ELK3 and enhanced its ubiquitination. Furthermore, ectopic expression of SPOP attenuated the ELK3 protein half-life, and SPOP depletion recovered the ELK3 protein level. Structurally, SPOP recognized the degron consensus sequence (^129^LRSTS^133^) in ELK3. Several studies have reported that mutations of the degron motif of substrates are found in cancer patients, which contributes to cancer development and/or drug resistance by inhibiting interactions with their E3 ligases and escaping degradation [19, 43, 44]. Thus, we analyzed the presence of mutations in ELK3 aa 129–133 using TCGA and COSMIC databases (https://cancer.sanger.ac.uk/cosmic). We found a T132M missense mutation of ELK3 and speculated that the mutation might interrupt ELK3 ubiquitination and subsequent degradation induced by SPOP, although we did not conduct biological experiments in this regard. Thus, further studies are needed to elucidate this idea.

CHKs are serine/threonine kinases and play an important role in signal transduction responses to DNA damage [45]. Activated CHKs phosphorylate multiple downstream substrates, such as p53, Brca1, and Cdc25C, which are associated with DNA repair, cell cycle arrest, apoptosis, and chromatin remodeling [46–48]. Generally, CHKs recognize the consensus sequence (R-X-X-S/T) for the phosphorylation of substrates [49]. Huy Q. Ta has previously demonstrated that CHK2 negatively regulates AR transcriptional activity and PCa cell growth. Moreover, CHK2 protein levels are inversely correlated with PCa grade [50]. Notably, CHK2 mutation or expression downregulation induces PCa survival in response to DNA damaging stimulus, such as ionizing radiation [51]. Here, we found that CHK1/2 phosphorylated ELK3 and enhanced the interaction between ELK3 and SPOP, thereby inducing SPOP-mediated ELK3 ubiquitination and subsequent degradation. Accordingly, CHK1/2 inhibition or gene silencing stabilizes the ELK3 protein. Therefore, our results suggest the possibility of a novel therapeutic strategy targeting the CHKs-ELK3-SPOP axis to eliminate PCa.

The physiological relevance of ELK3 in PCa malignance in humans is suggested by our current research. We collected 117 PCa tissues and analyzed them using IHC. Automatic IHC analysis using the TissueFAXS IHC analysis computer system demonstrated that ~85% of human PCa tissues showed higher ELK3 protein levels than the normal tissue area. Moreover, since c-Fos, a downstream transcription factor governed by the expression of ELK3 [13], is critical in forming the AP-1 complex, which is known to involve ~50–60% of gene expression in eukaryotic cells, we believe that ELK3 protein content control may provide a critical clue for finding a new strategy to resolve PCa malignance. Although docetaxel induces ELK3 protein degradation through SPOP-mediated ubiquitination, PCa-associated SPOP mutants did not disrupt ELK3 protein abundance, even under docetaxel treatment. Since SPOP mutations have been identified in ~4.4–28.6% of PCa patients [52], research on ELK3 governing the regulation of protein amount and activity is invaluable. This notion was supported by *ELK3* depletion promoting docetaxel-induced cell death in PCa cells containing SPOP mutants via cellular apoptosis [12]. Therefore, we suggest that ELK3 targeting agents, such as transcription factors–proteolysis targeting chimeras, which enable specific targeting and degrading of transcription factors via the ubiquitin-dependent proteasome [53], could be a promising therapeutic strategy for the treatment of PCa harboring SPOP mutations. In summary, our results not only elucidate the oncogenic function of ELK3 regulated by SPOP-mediated ELK3 ubiquitination and degradation, but also verify that aberrant accumulation of ELK3 by SPOP mutations could induce resistance to docetaxel. Although earlier detection of prostate cancer has showed ~100% of 5-year survival rate, people with spread prostate cancer to other parts of the body dramatically dropped to 31% of 5-year survival rate. Moreover, SPOP mutation has been identified in ~15% of prostate cancer patients, SPOP-ELK3 protein stability regulation may suggest a key clue to improve prostate cancer mortality.

### Supplementary information

Supplementary information

whole blots for the Western blotting

## Data Availability

The datasets used and/or analyzed during this study can be accessed from the corresponding author upon request.
