# Supplementary material for: ELK3 destabilization by speckle-type POZ protein suppresses prostate cancer progression and docetaxel resistance
Source: Cell Death Dis. 2024 Apr 17;15(4):274. doi: 10.1038/s41419-024-06647-0 (PMC11024157; doi:10.1038/s41419-024-06647-0)
Supplement: Supplementary file 1 — Supplementary information [file 41419_2024_6647_MOESM1_ESM.pdf]

[Supplementary information]

ELK3 destabilization by speckle-type POZ protein suppresses prostate cancer progression and docetaxel resistance

Cheol-Jung Lee[1,2], Heejung Lee[3], Seo Ree Kim[4], Soo-Bin Nam[1,2], Ga-Eun Lee[1], Kyeong Eun Yang[2], Guk Jin Lee[4], Sang Hoon Chun[4], Han Chang Kang[1,6], Joo Young Lee[1,6], Hye Suk Lee[1,6], Sung-Jun Cho[5], and Yong-Yeon Cho[1,6,*]

Content

1. Supplementary Materials and Methods: cell proliferation and viability assays, immunoblotting and immunoprecipitation, luciferase reporter assay, and migration assay

2. Supplementary Figure legends: 6 Figures

1. Supplementary Materials and Methods

Cell proliferation and viability assays

To explore the effect of SPOP and ELK3 on cell proliferation, 22Rv1 cells (2×10^3) stably depleted SPOP and/or ELK3 were seeded into 96-well plates. For the evaluation of cell viability, 22Rv1 cells (2×10^4) stably expressing SPOP-wt or -mutants were seeded into 96-well plates and treated with 1 μM of docetaxel for 48 h. All plates were supplemented with 10 μL of cell counting kit-8 (cat. #: CK04, Dojindo, Kumamoto, Japan) solution and followed by 1 h incubation at 37°C in a 5% CO_2 humidified atmosphere. The cell density was evaluated by measuring the absorbance at 450 nm using an xMark microplate spectrophotometer (Bio-Rad Laboratories, Hercules, CA, USA).

Immunoblotting and immunoprecipitation

Cell lysates obtained by disruption of the cells using lysis buffer (50 mM Tris-Cl, pH 7.5, 120 mM NaCl, 0.5% NP-40) supplemented with protease and phosphatase inhibitors were used to visualize specific proteins by WB and immunoprecipitation (IP). In brief, equal amounts of cell lysates (generally 30 μg for WB; 200–500 μg for IP) were used. The IP of Xpress-, Flag-, Myc-, or His-tagged protein was conducted by coupling each cell lysate, tag-specific primary antibodies, and protein G agarose beads at 4°C overnight. The beads were washed five times in NETN buffer (20 mM Tris-Cl, pH 8.0, 100 mM NaCl, 0.5% NP-40, 1 mM EDTA), supplemented with 6× sample buffer, boiled at 95 °C for 5 min, and then centrifuged to obtain the IP precipitates. The precipitates and whole-cell lysates were resolved by SDS-PAGE and transferred to PVDF membranes. The membranes were blocked with 5% skim milk/TBST blocking buffer for 1 h at room temperature, hybridized with the indicated primary antibodies at 4°C overnight, and hybridized with the indicated secondary antibodies conjugated with horseradish peroxidase (HRP). The target proteins were visualized by enhanced chemiluminescence (Amersham Biosciences, Piscataway, NJ, USA) using a Chemidoc XRS imager system (Bio-Rad Laboratories).

Luciferase reporter assay

A *c-fos* promoter luciferase reporter assay was conducted to measure the change in ELK3 activity. In brief, HEK293T cells were transiently co-transfected with a combination of *c-fos*-promoter luciferase reporter plasmid, ELK3-wt, SPOP, ELK3-wt, ELK3-ΔDeg1, sh-mock, and sh-SPOP, and cultured for 24–36 h. The cell lysates were obtained by cell lysis buffer (Promega, Madison, WI, USA) and used to measure firefly luciferase activity using a

VIXTOR X3 luminometer (PerkinElmer Inc., Waltham, MA). The firefly luciferase activity was normalized with phRL-Renilla luciferase activity, which was used as an internal control for equal transfection.

Migration assay

22Rv1 and DU145 cells (2.5×10^4) were stably depleted of SPOP, ELK3, or SPOP and ELK3 were seeded onto the transwell filters (Corning, NY, USA), which were supplemented with FBS-free medium in the upper chamber and cultured in 24-well chamber plates supplemented with complete medium in the bottom chamber for 24 h. The non-migrated cells in the upper side of the transwell filters were wiped out using ear cotton plugs. The migrated cells were fixed with 4% formaldehyde, permeabilized by methanol treatment, and stained with 0.1% crystal violet. The stained cells were photographed under an inverted microscope. The migrated area was measured using the National Institute of Health’s Image J computer program (version 1.53k).

2. Supplementary Figures

Interaction type	amino acid ELK3	amino acid SPOP	Distance (A)
Hydrogen bond	Arg 130	Asp 144	1.94
	Lys 113	Lys 110	2.88
	Ser 133	Lys 115	1.95
	Glu 117	Leu 150	2.67
	Arg 119	Leu 143	2.19
	Ser 140	Lys 135	2.06
	Ser 144	Glu 118	2.23
	Phe 146	Glu 118	2.45
	Glu 89	Lys 135	3.35
	Arg 130	Asp 144	3.26
	Glu 352	Arg 543	2.60
Electrostatic interaction	Glu 136	Lys 115	4.51
	Glu 89	Lys 135	3.68
	Arg 119	Glu 145	4.42
	Arg 130	Glu 145	5.20
	Phe 146	Lys 101	3.35
	Phe 146	Glu 118	4.57
Hydrophobic interaction	Leu 151	Ile 27	5.19
	Pro 88	Lys 135	5.30
	Ala 121	Ala 146	3.90
	Leu 151	Val 164	4.36
	Leu 142	Phe 133	5.36
	Arg 134	Phe 141	5.22
	Tyr 137	Lys 115	5.21
	Phe 146	Lys 101	4.33

Supplementary Fig. S1 Analysis of SPOP-ELK3 interaction interface. The interaction interface was analyzed by Discovery Studio software program and the interaction types were shown in table. Amino acids marked in blue indicate amino acids included in the SPOP degron motif.

Human 124-HGLAALRSTSRNEYI-138
Chimpanzee 124-HGLAALKSTSRNEYI-138
Monkey 124-HGLSALKSTSRNEYI-138
Chicken 124-HGLAALKSTSRNEYI-138
Chameleon 124-HGLSVLKSTSRNEYI-138

CHK1/2 phospho-motif : R/K-x-x-S/T

Supplementary Fig. S2. ELK3 harbors conserved amino acid motif for CHK1/2-mediated phosphorylation.

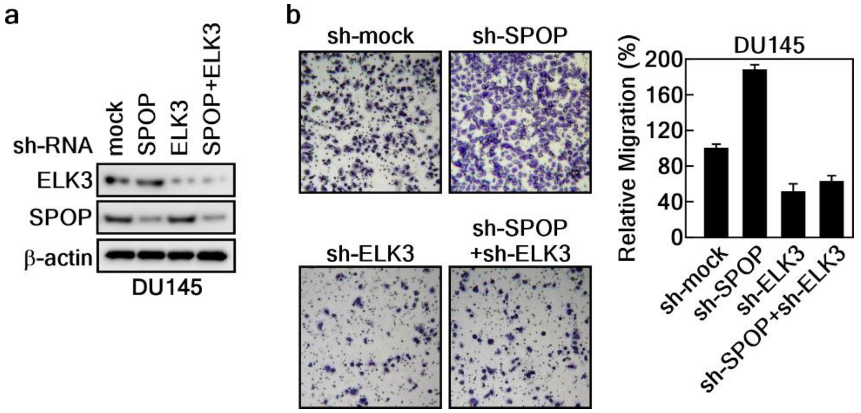

Supplementary Fig. S3 SPOP-mediated ELK3 degradation suppresses prostate cancer migration. a knockdown efficiency of ELK3 and SPOP were evaluated by WB in DU145 cells. **b** Regulatory effect of SPOP and ELK3 on prostate cancer cell migration was evaluated by cell migration assay.

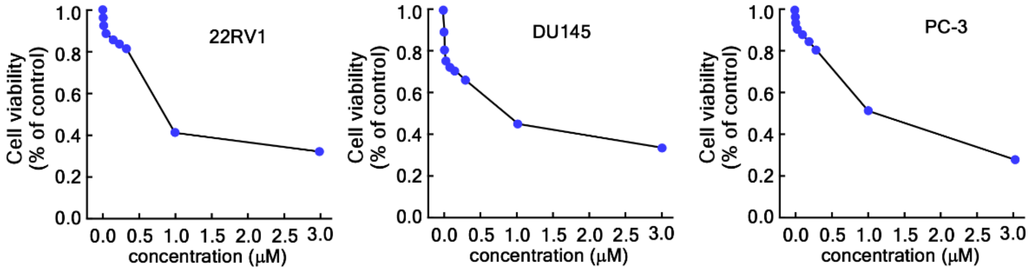

Supplementary Fig. S4 Evaluation of IC50 for docetaxel in PCa cells. PCa cells, including 22RV1, DU145, and PC-3, were used to determine the docetaxel sensitivity by measuring the IC_{50} values using CCK-8 assay kit.

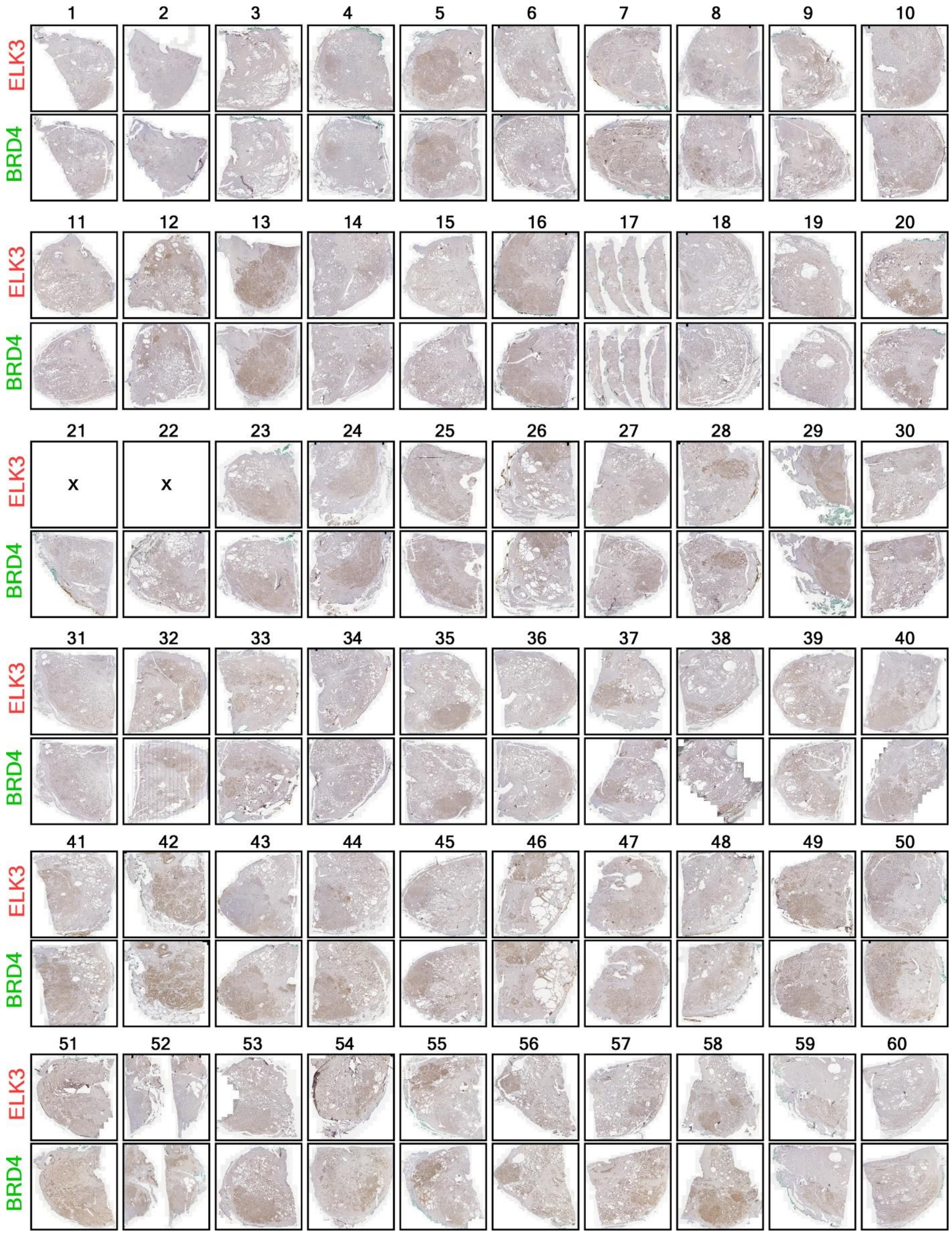
ELK3
BRD4
1
2
3
4
5
6
7
8
9
10
11
12
13
14
15
16
17
18
19
20
21
22
23
24
25
26
27
28
29
30
31
32
33
34
35
36
37
38
39
40
41
42
43
44
45
46
47
48
49
50
51
52
53
54
55
56
57
58
59
60
x
x

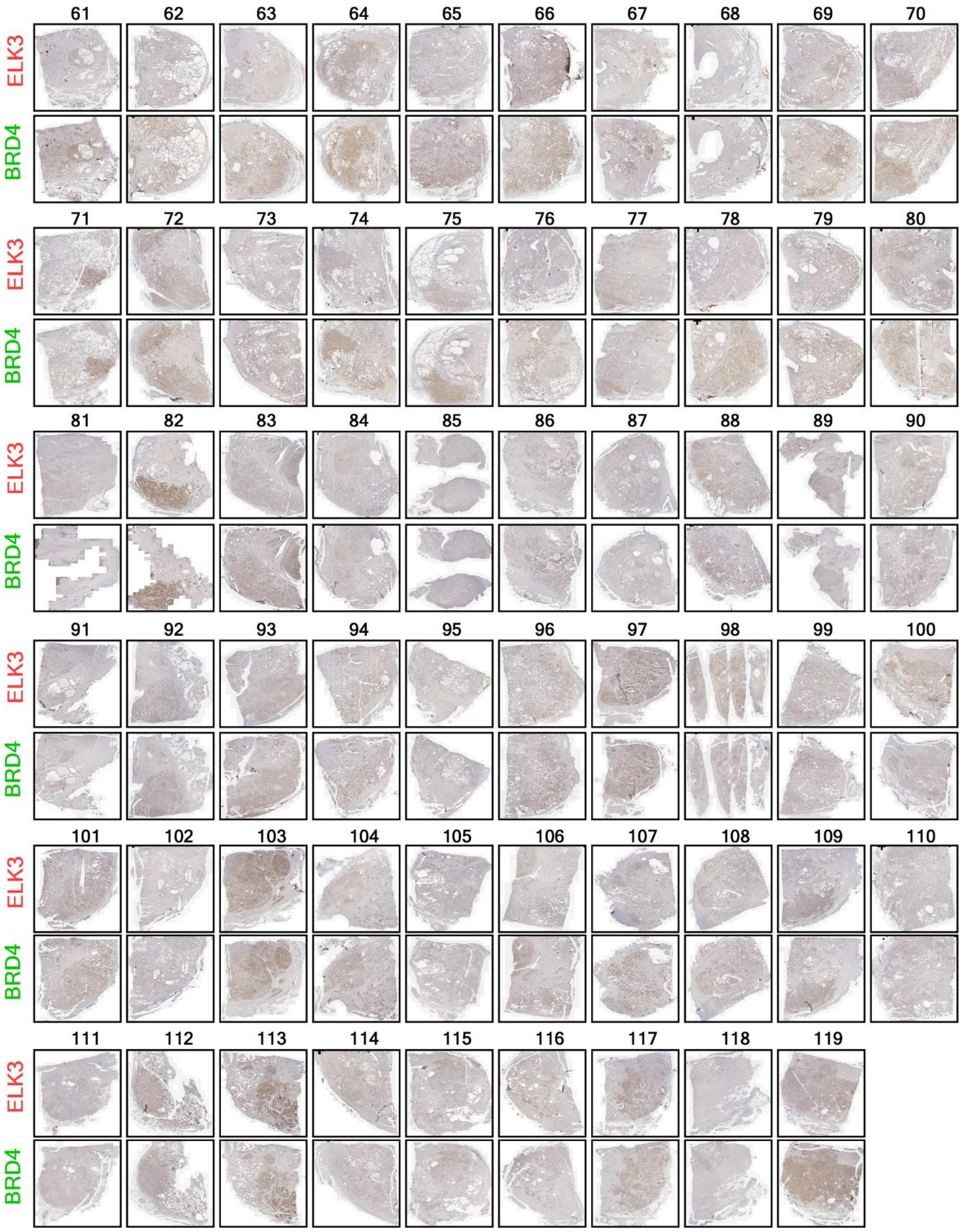

Supplementary Fig. S5 Original IHC images of PCa tissues. PCa tissues (n=117) were stained with ELK3 or BRD4 specific antibodies.

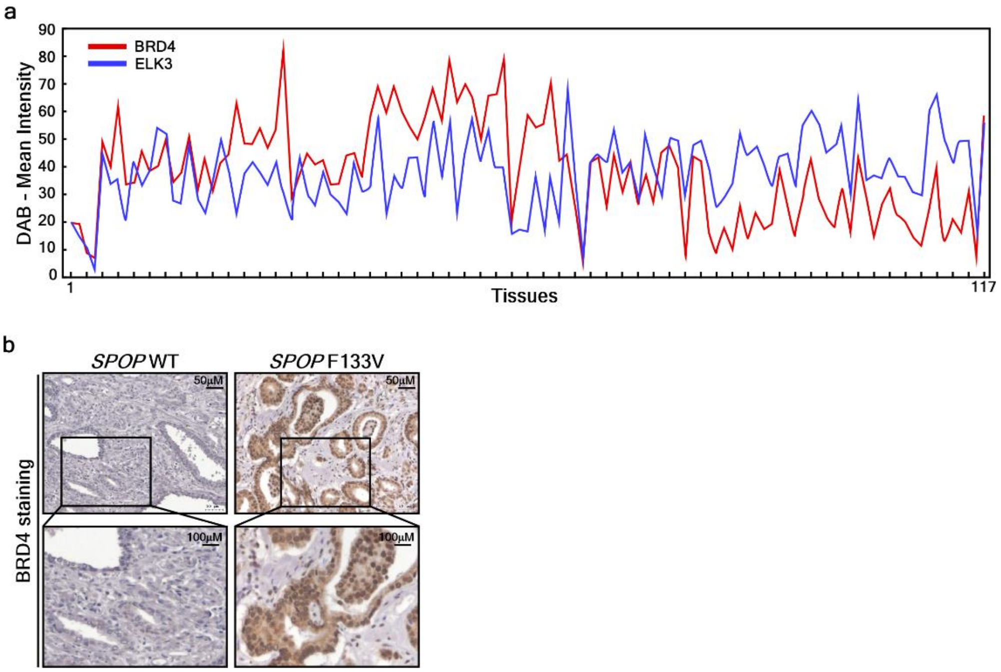

Supplementary Fig. S6 Protein expressions in PCa patient's tissues. a Comparison of BRD4 and ELK3 expression in PCa tissues. The PCa tissues were stained with BRD4 or ELK3 specific antibodies using IHC experiment. The protein expression was measured and analyzed by TissueFAXS. **b** Comparison of BRD4 expression in PCa tissues harboring *SPOP*-WT or *SPOP* mutation (F133V). The PCa tissue was stained with BRD4 specific antibody using IHC experiment. The protein expression was measured and analyzed by TissueFAXS.
