# Supplementary figures and images for: ELK3 destabilization by speckle-type POZ protein suppresses prostate cancer progression and docetaxel resistance
Source: Cell Death Dis. 2024 Apr 17;15(4):274. doi: 10.1038/s41419-024-06647-0 (PMC11024157; doi:10.1038/s41419-024-06647-0)

Fig.1

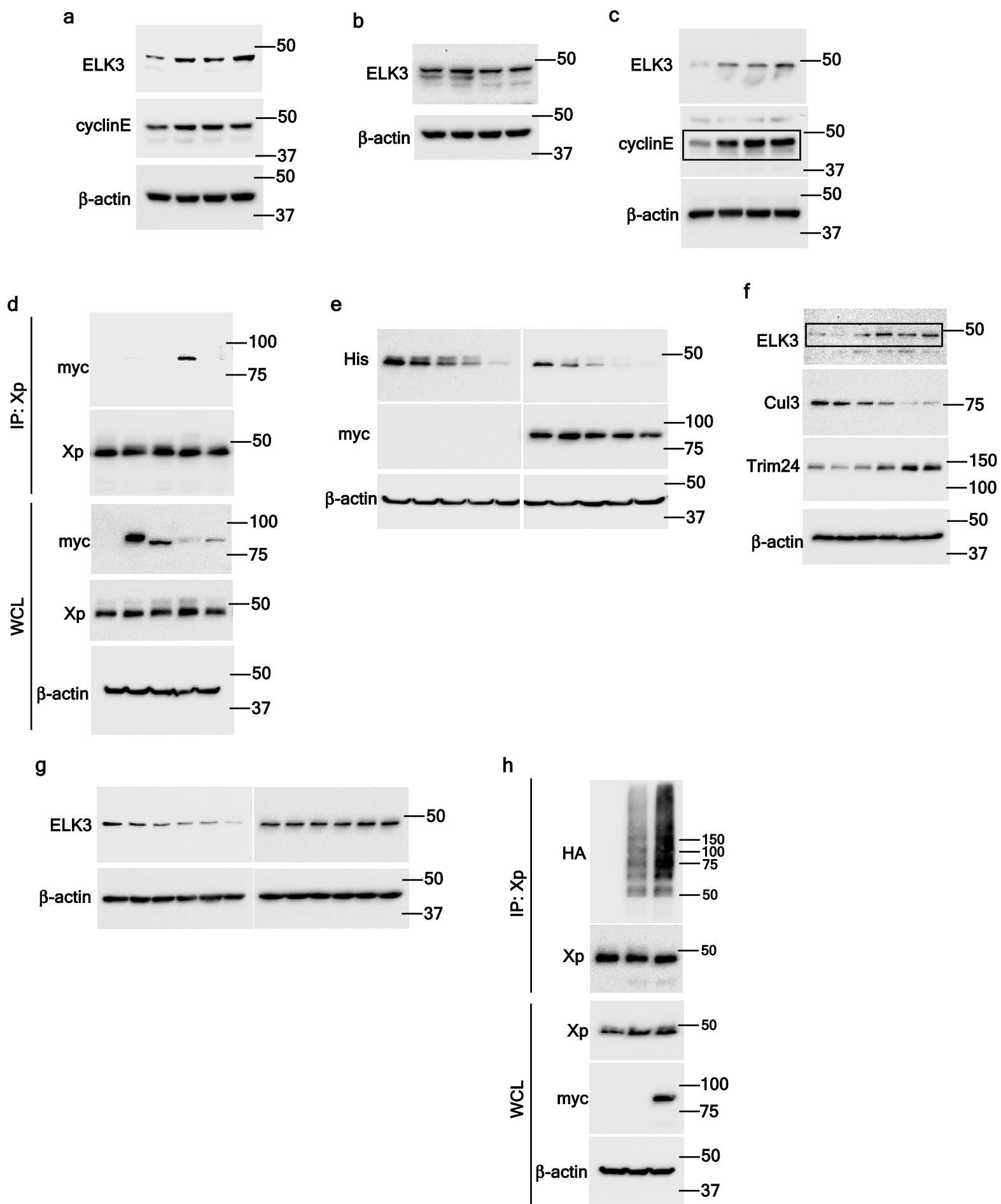

Fig.2

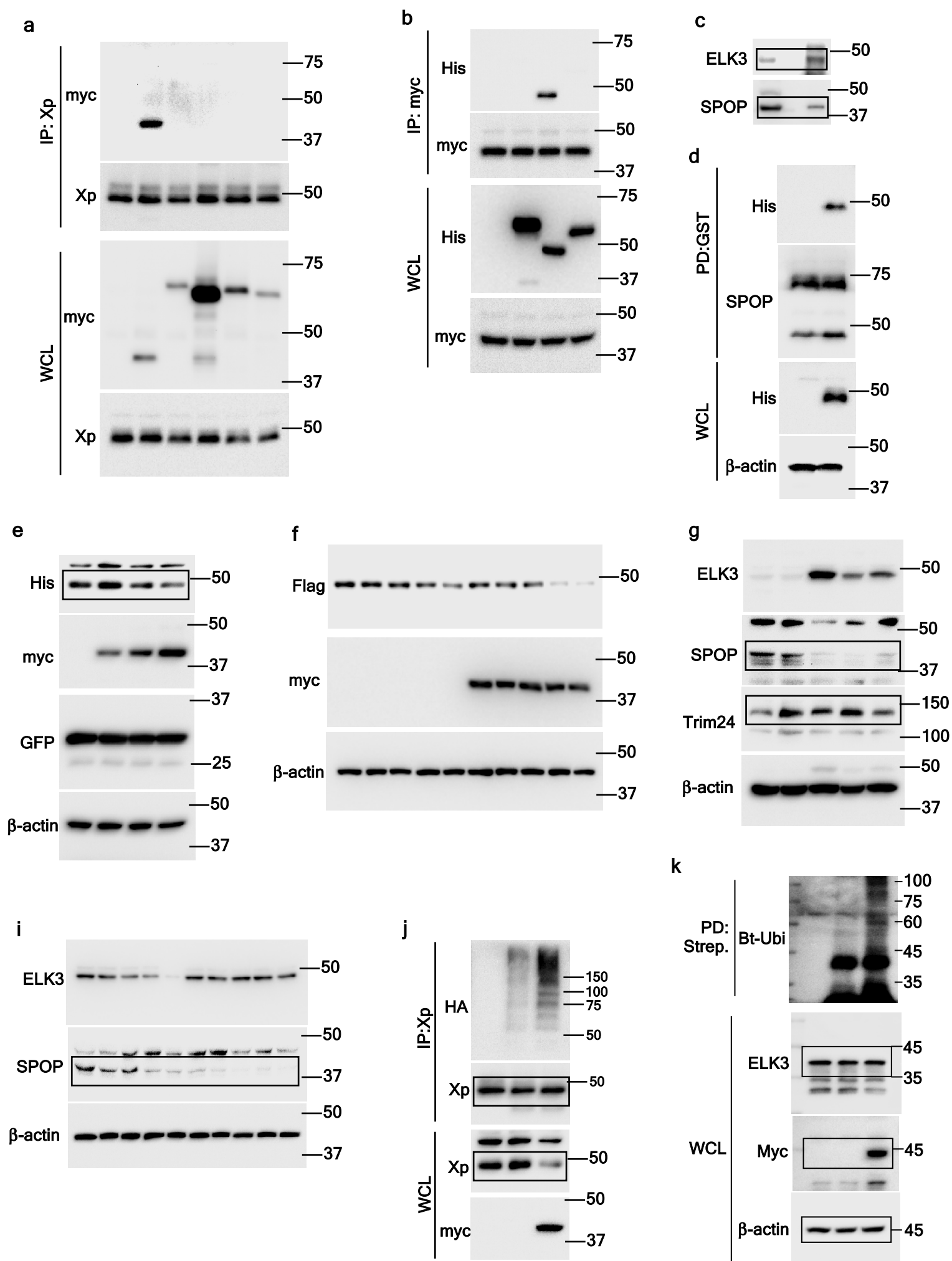

Fig.3

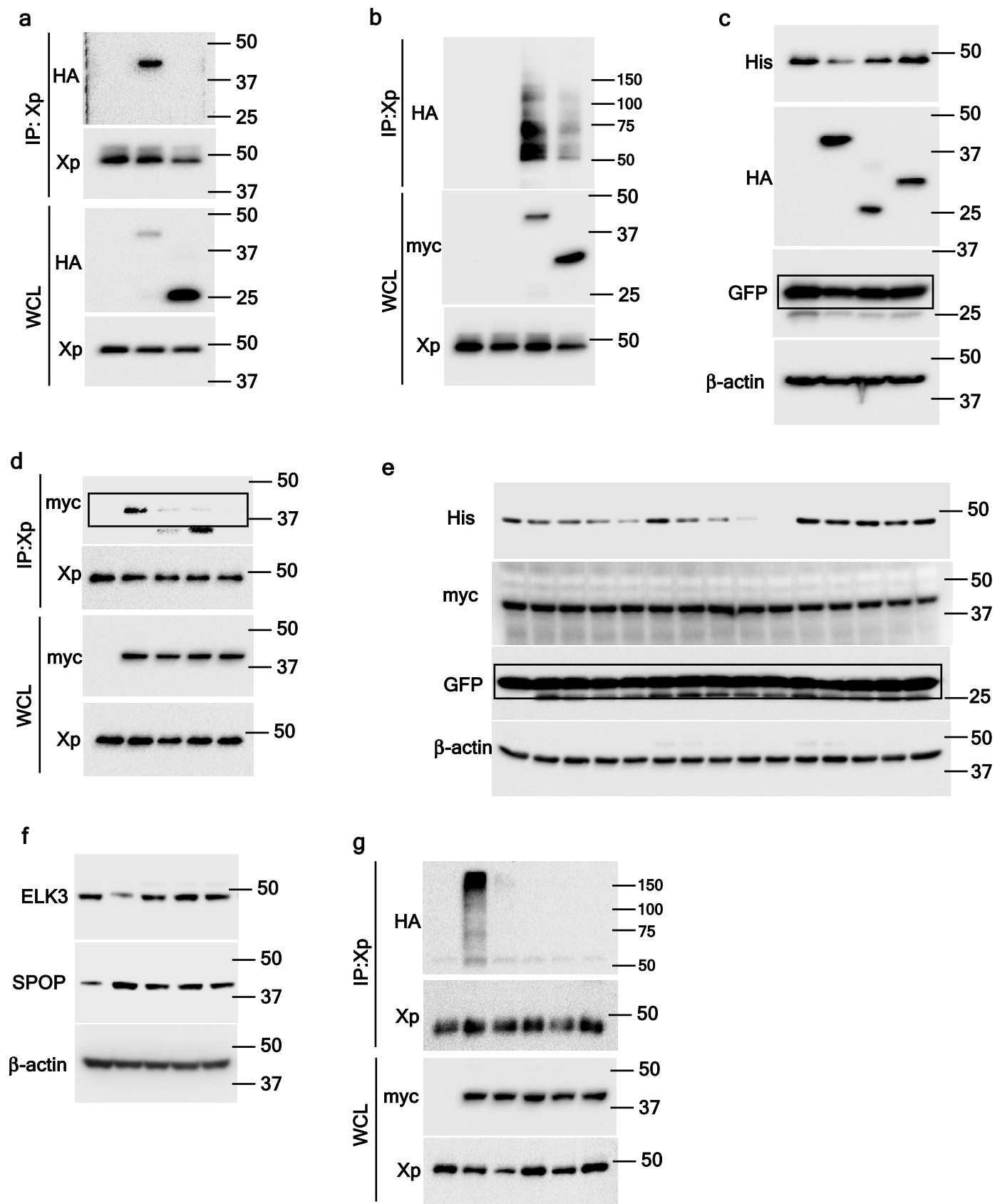

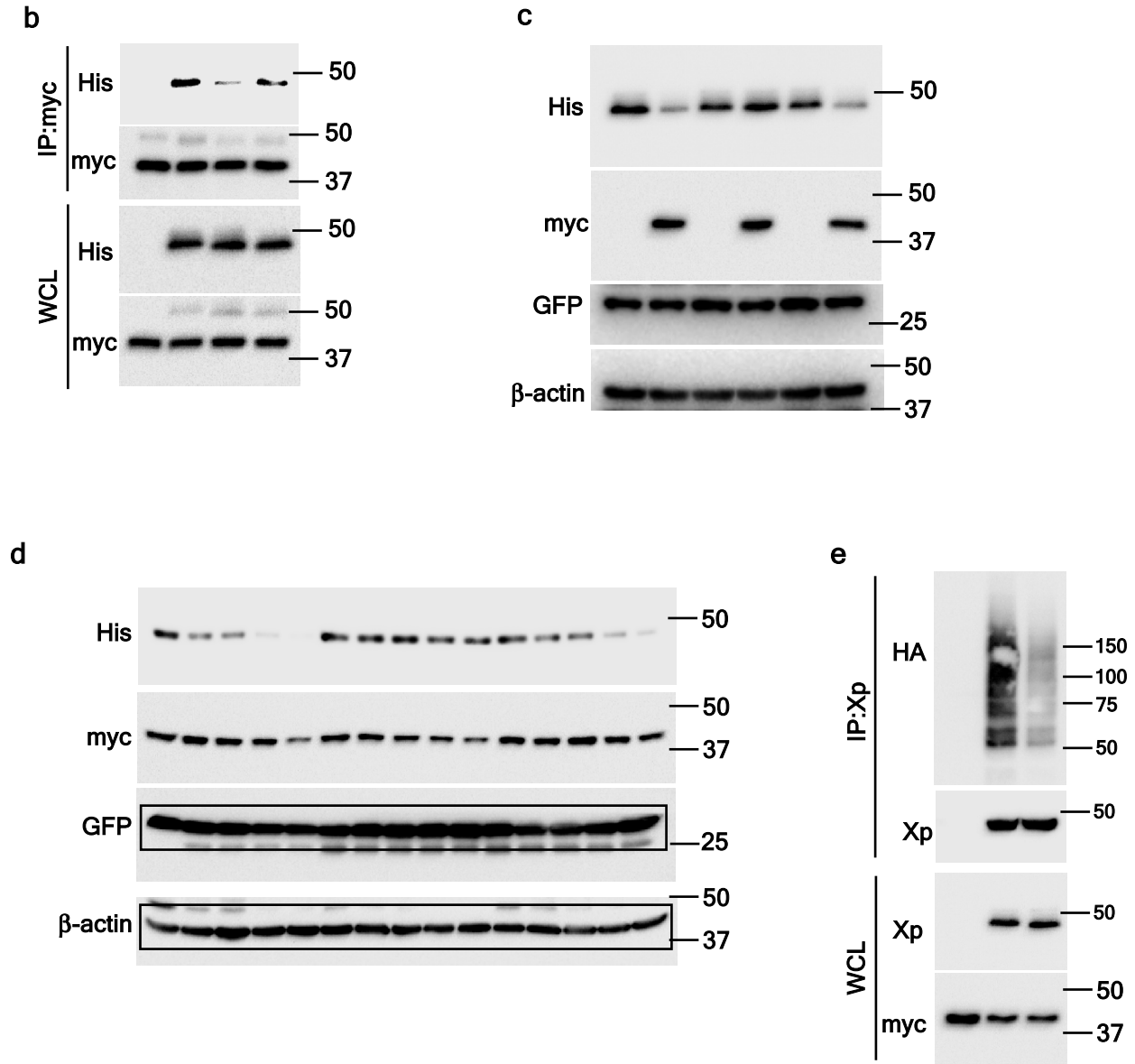

Fig.5

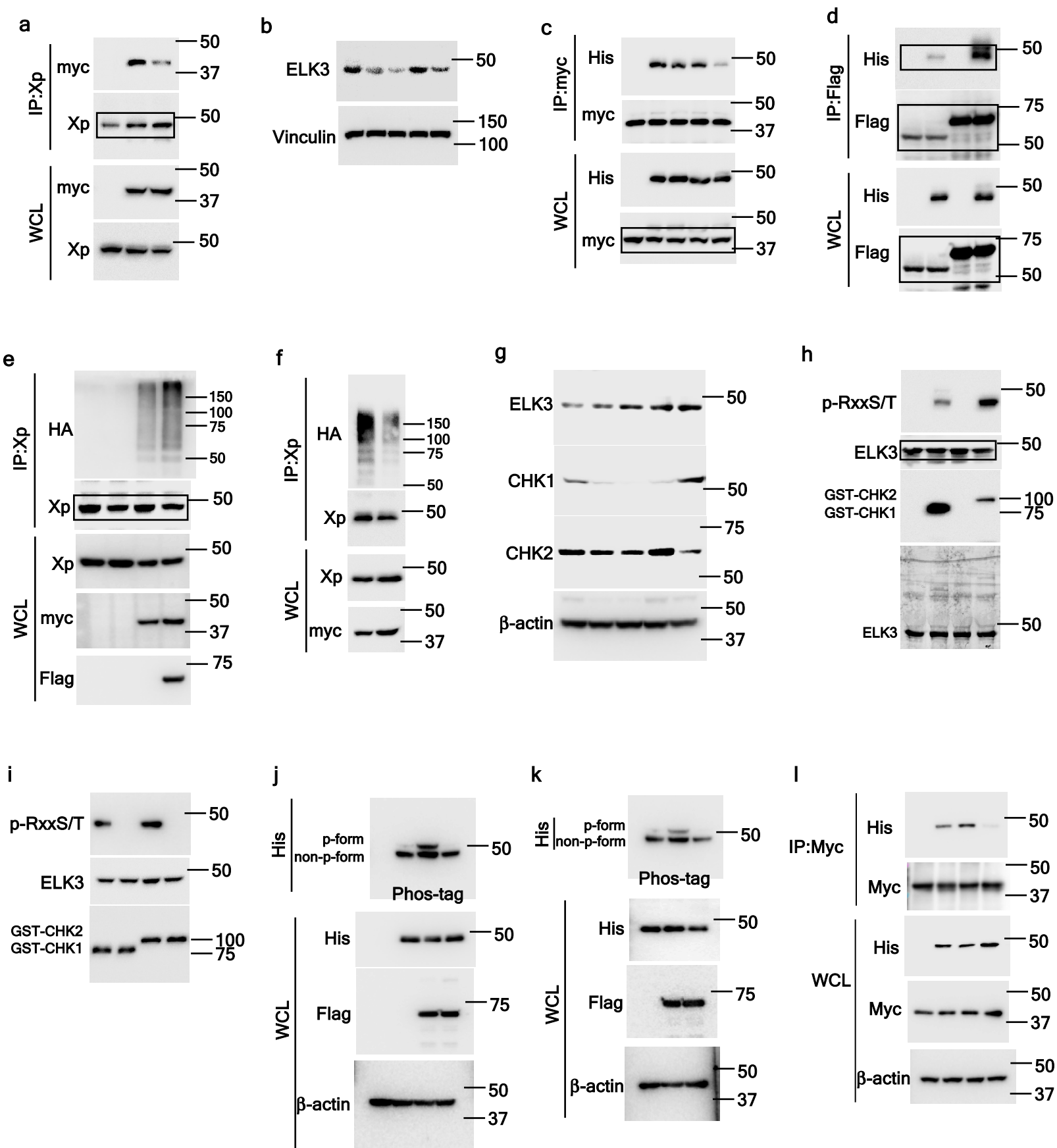

d

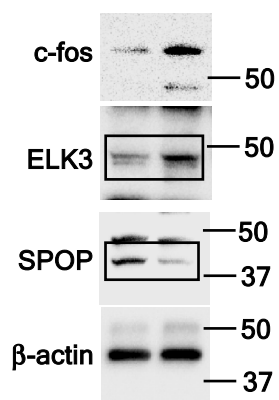

e

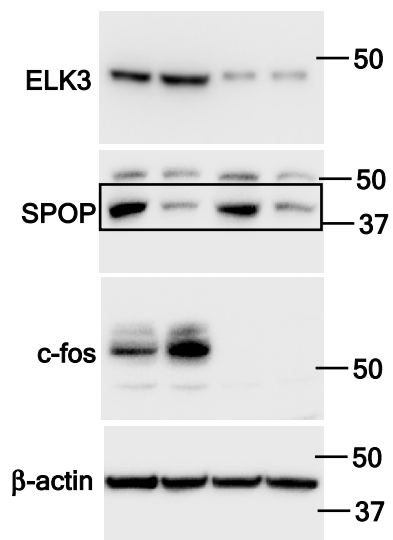

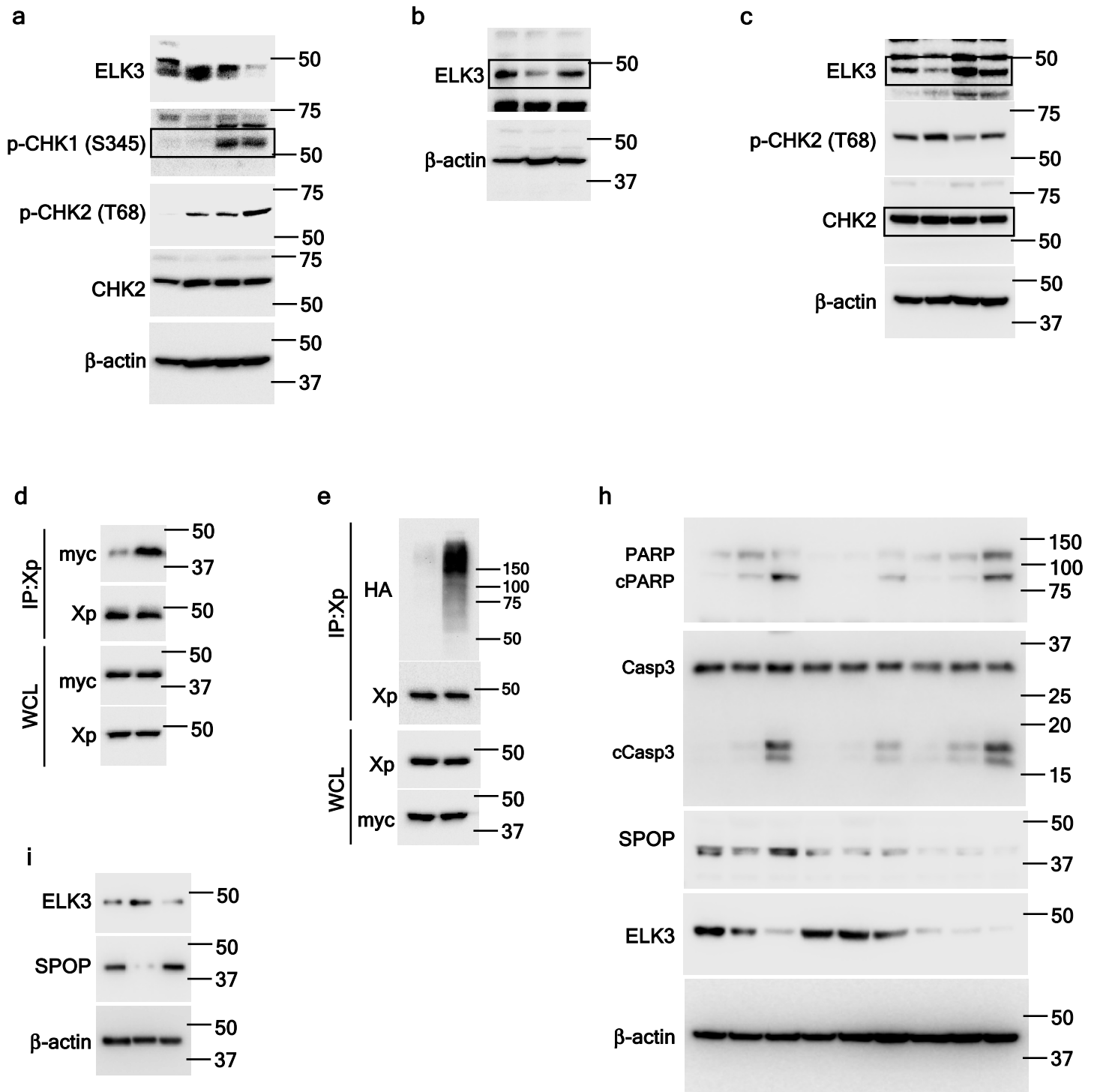

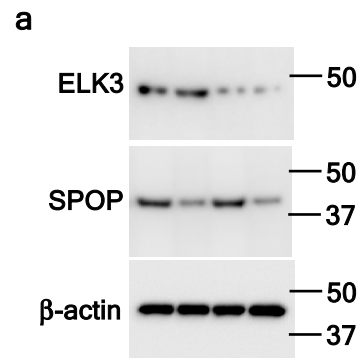

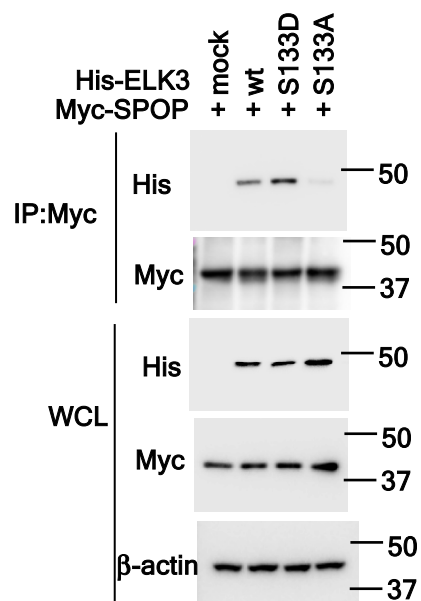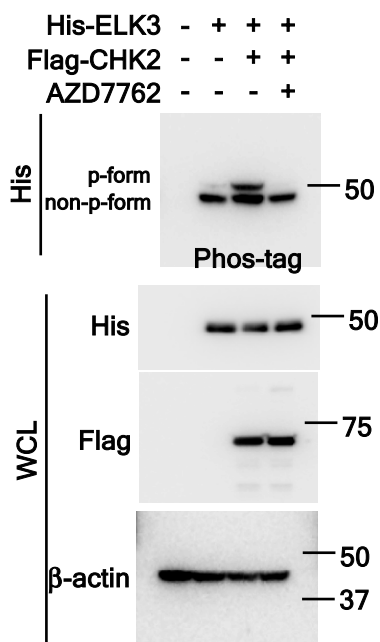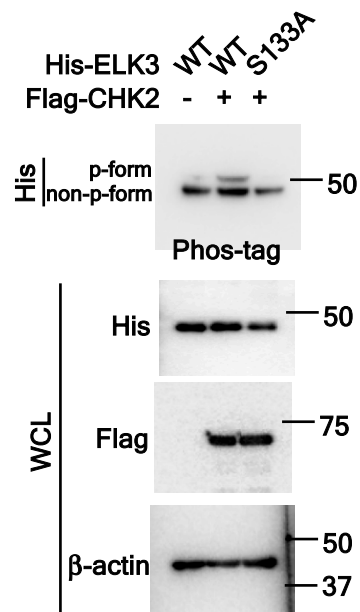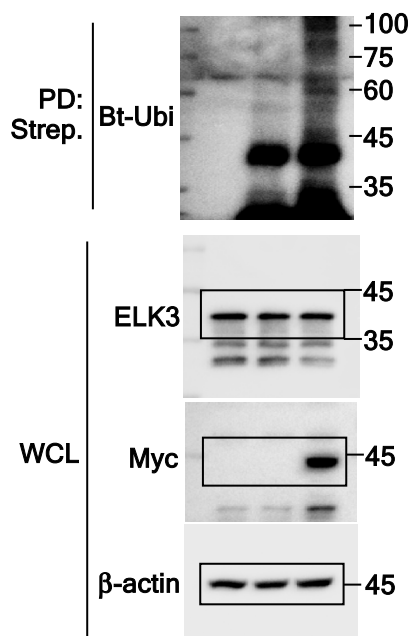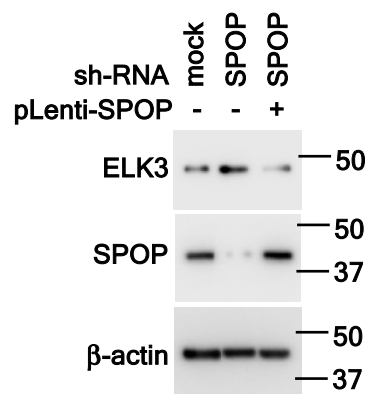

Supplement: Supplementary file 2 — whole blots for the Western blotting [file 41419_2024_6647_MOESM2_ESM.pdf]
